# Supplementary material for: Split selectable markers
Source: Nat Commun. 2019 Oct 31;10:4968. doi: 10.1038/s41467-019-12891-2 (PMC6823381; doi:10.1038/s41467-019-12891-2)
Supplement: Supplementary file 1 — Supplementary Information [file 41467_2019_12891_MOESM1_ESM.pdf]

Split Selectable Markers

Supplementary Information

Jillette and Du *et al.*

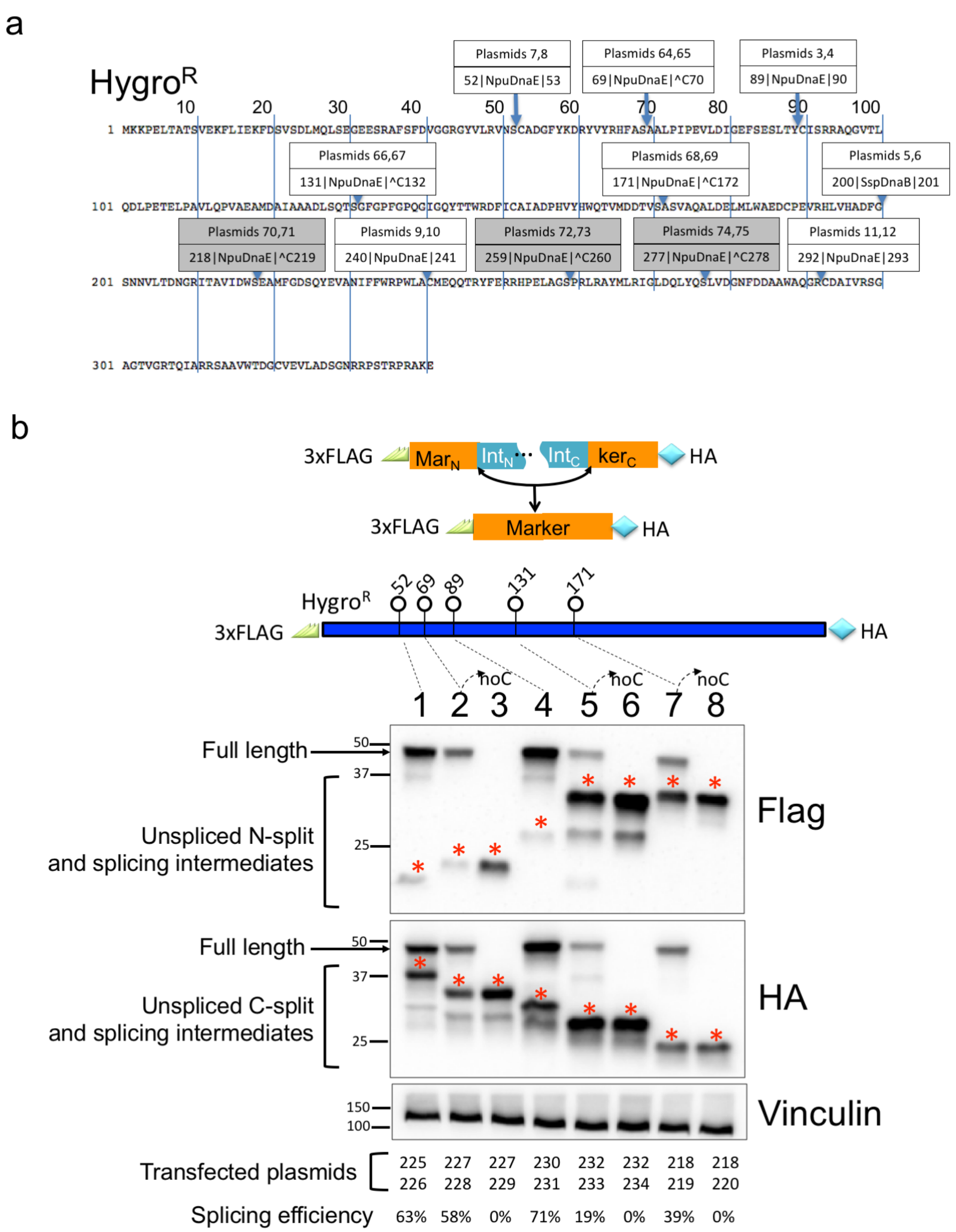

Supplementary figure 1. (a) Split points for the Hygromycin resistance (Hygro^R) protein. Amino acid sequence of Hygro^R protein is shown with clouds labeling the split points. Within the label, the top row indicates the plasmid numbers corresponding to Supplementary Table 1. The bottom row indicates the residue number of the last amino acid in the N-terminal fragment, the species of the intein used, and the residue number of the first amino acid in the C-terminal fragment. "^C" indicates an insertion of a Cysteine. **(b)** Western blot analysis of protein trans-splicing of Hygromycin

markertrons. A N-markertron is N-terminally tagged with 3xFLAG epitope while a C-marketron is C-terminally tagged with HA epitope. Western blot photograph of lysates from HEK293T cells transfected with the indicated N- and C-markertrons, using the indicated antibodies (on the right). The bands corresponding to the N-split, C-split or reconstituted marker proteins are indicated on the left. Asterisks mark unspliced markertron fragments. Vinculin serves as equal-loading control.

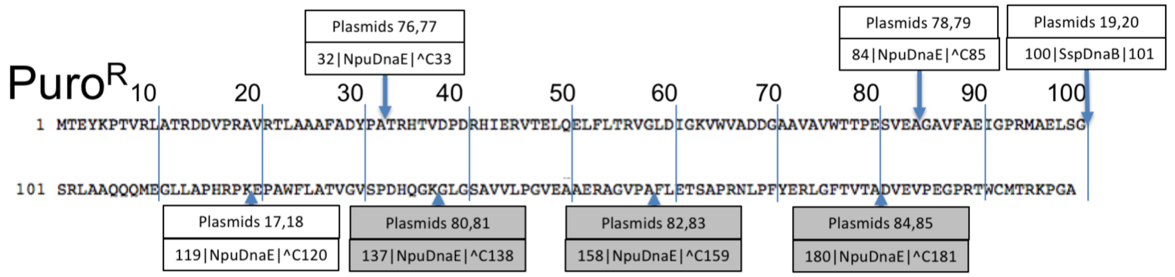

Supplementary figure 2. Split points for the Puromycin resistance (Puro^R) protein. Amino acid sequence of Puro^R is shown with clouds labeling the split points. Within the label, the top row indicates the plasmid numbers corresponding to Supplementary Table 1. The bottom row indicates the residue number of the last amino acid in the N-terminal fragment, the species of the intein used, and the residue number of the first amino acid in the C-terminal fragment. "^C" indicates an insertion of a Cysteine.

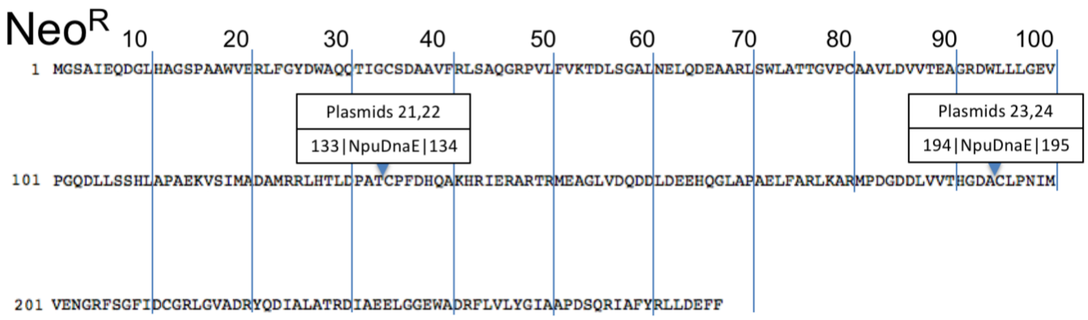

Supplementary figure 3. Split points for the Neomycin/G418 resistance (Neo^R) protein. Amino acid sequence of Neo^R is shown with clouds labeling the split points. Within the label, the top row indicates the plasmid numbers corresponding to Supplementary Table 1. The bottom row indicates the residue number of the last amino acid in the N-terminal fragment, the species of the intein used, and the residue number of the first amino acid in the C-terminal fragment.

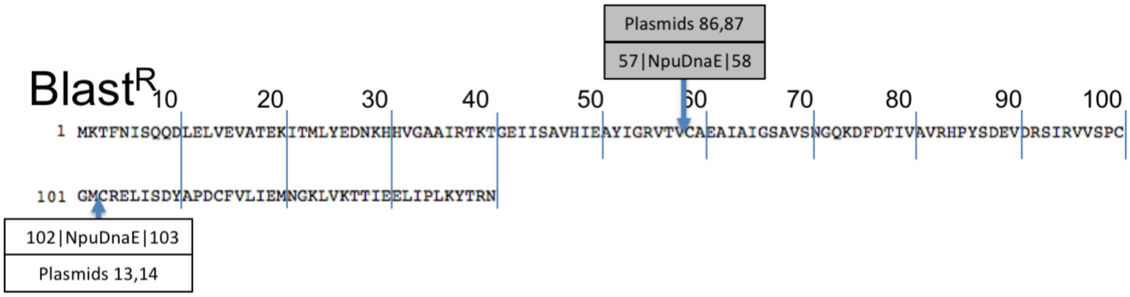

Supplementary figure 4. Split points for the Blasticidin resistance (Blast^R) protein. Amino acid sequence of Blast^R is presented with clouds labeling the split. Within the label, the top row indicates the plasmid numbers corresponding to Supplementary Table 1. The bottom row indicates the residue number of the last amino acid in the N-terminal fragment, the species of the intein used, and the residue number of the first amino acid in the C-terminal fragment.

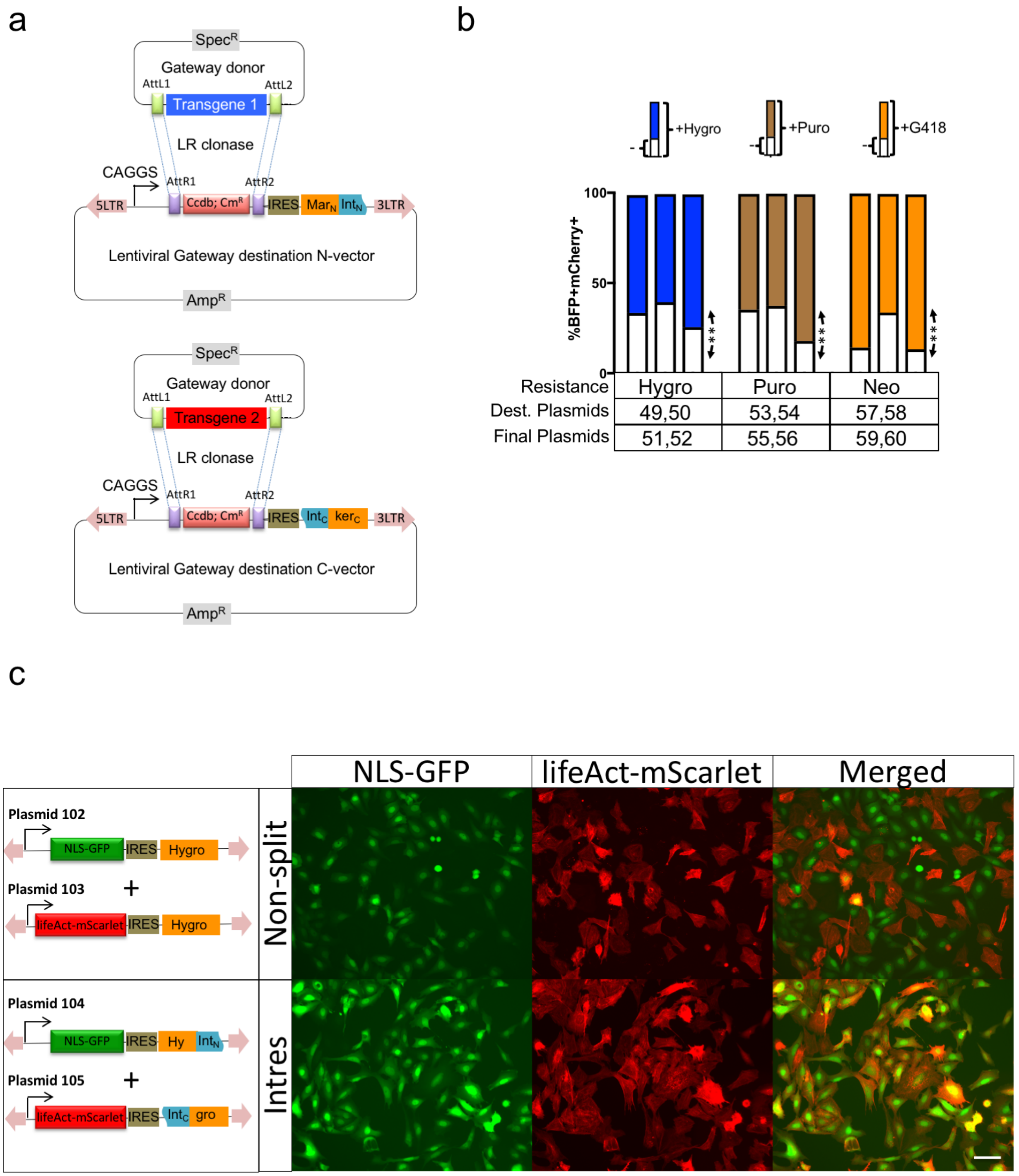

Supplementary figure 5. Gateway-compatible lentiviral destination vectors with 2-split Intres markers. (a) Gateway-compatible lentiviral destination vector kits for each split Intres marker consists of an N-vector and C-vector. N-vector contains viral LTRs, CAGGS promoter, Gateway destination cloning cassette that allows LR clonase-mediated recombination of the Gateway donor vector carrying transgenes, followed by an internal ribosomal entry site (IRES) that allows polycistronic expression of the N-markertron. Similarly, the C-vector contains the C-markertron and allows recombination of another transgene. (b) TagBFP (as transgene 1) and mCherry (as transgene 2) were cloned into the 2-split Intres plasmids by Gateway recombination and delivered to cells by lentiviral transduction, followed by antibiotic

selection and flow cytometry analysis. Column plot shows the percentage of BFP+mCherry+ double-positive cells in the selective culture from the 2-split Hygromycin (Hygro, white+blue column), Puromycin (Puro, white+brown column), and Neomycin (Neo, white+orange column) experiments versus their corresponding non-selective cultures (white portion of each column). Vertical asterisks indicate statistical significance by paired two-sided t-test on the percentages of double-positive cells in the selected cultures vs non-selected cultures within each transfection group (n.s., non-significant; *p<0.05, **p<0.01,***p<0.001). (c) NLS-GFP (as transgene 1) that labels the nucleus with GFP fluorescence and lifeAct-mScarlet (as transgene 2) that labels F-actin with mScarlet fluorescence were recombined into lentiviral vectors expressing full non-split $Hygro^R$ or lentiviral vectors with 2-split Hygro Intres genes and used to transduce U2OS cells to make dual-label cells. Representative fluorescence microscopic images show GFP, mScarlet and merged channels of cells after Hygromycin selection for two weeks. Scale bar: 20µm.

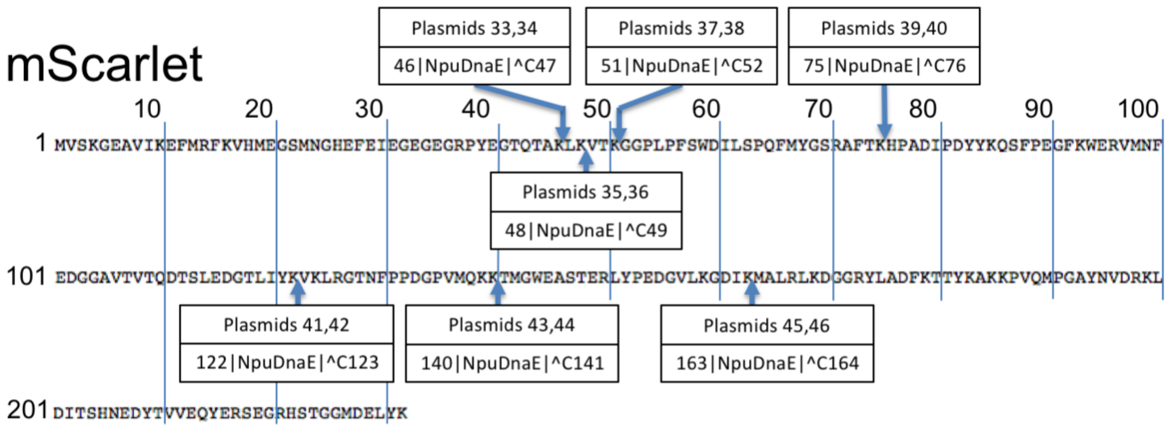

Supplementary figure 6. Split points for mScarlet fluorescent protein. Amino acid sequence of the mScarlet gene is shown with clouds labeling the split points. Within the label, the top row indicates the plasmid numbers corresponding to Supplementary Table 1. The bottom row indicates the residue number of the last amino acid in the N-terminal fragment, the species of the intein used, and the residue number of the first amino acid in the C-terminal fragment. “^C” indicates an insertion of a Cysteine.

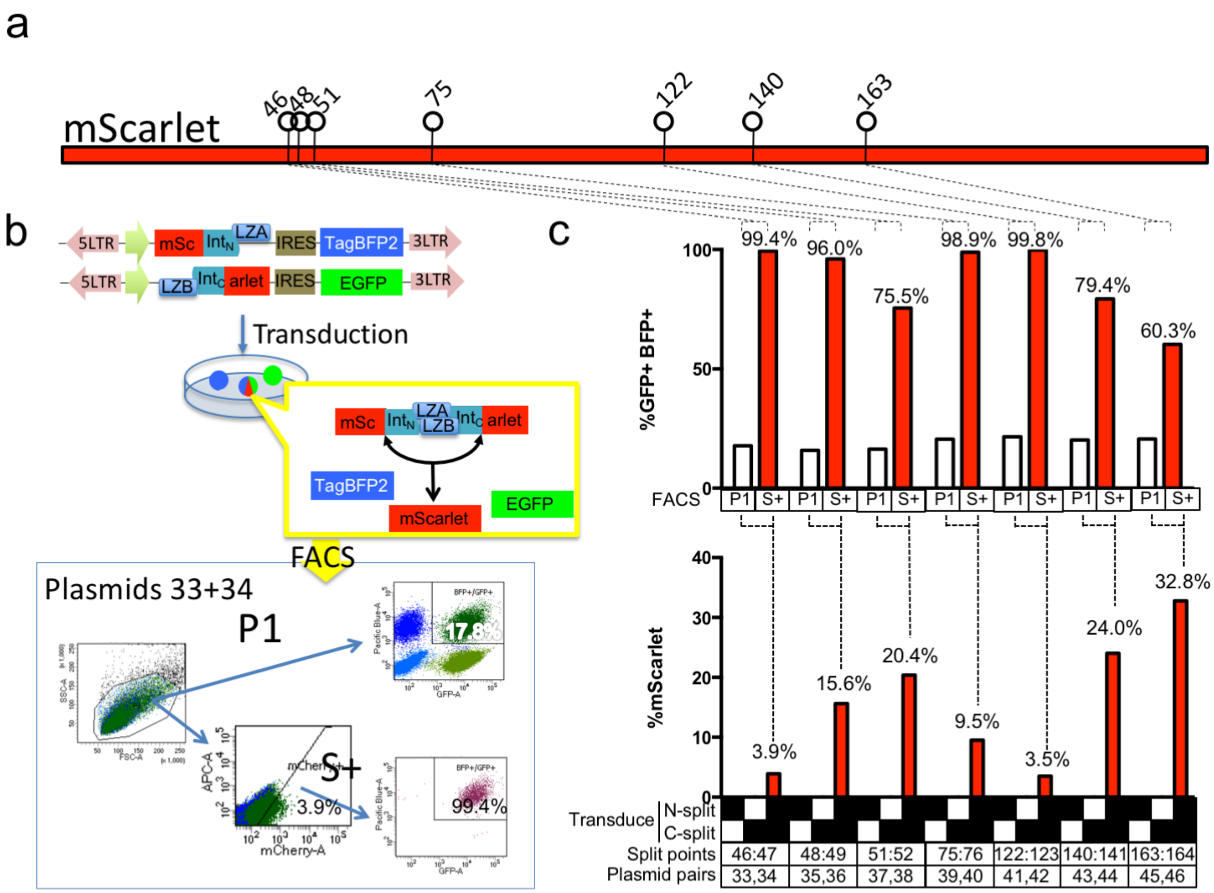

Supplementary figure 7. Split mScarlet for fluorescence-mediated co-selection of two separate transgenic vectors. (a) 2-split mScarlet proteins. Top schematic shows the split points tested for mScarlet. The last residue of the N-terminal fragment is indicated on top of the lollipops. (b) To screen for NpuDnaE intein-compatible split points for mScarlet, we identified potential split points according to the junctional requirement for the *Npu*DnaE intein, then cloned the corresponding N-terminal and C-terminal fragments to the split inteins scaffolds on lentiviral vectors equipped with TagBFP or EGFP fluorescent proteins, which serve as our test transgenes to evaluate the selection efficiency. These are delivered into cells via lentiviral transduction. Cells with both lentiviruses contain the necessary protein splicing machinery and mScarlet fragments to reconstitute the full-length mScarlet fluorescent protein, as well as express both TagBFP and EGFP transgenes. Cells were subjected to flow cytometry analysis. Boxed schematic shows an example of FACS analysis of the plasmid pair 33+34. P1 population was gated for forward scatter and side scatter for live single cells. From those, 17.8% of cells are double positive for TagBFP and EGFP transgenes. When the P1 cells were further gated for mScarlet-positive (mCherry channel), 99.4% of cells are double positive for TagBFP and EGFP. (c) The column plot below shows the percentage of mScarlet-positive cells for each of the indicated split points. The column plot above shows the percentage of BFP+EGFP+ cells among the P1 cells (white columns) and the mScarlet-positive subset of P1 cells (red columns).

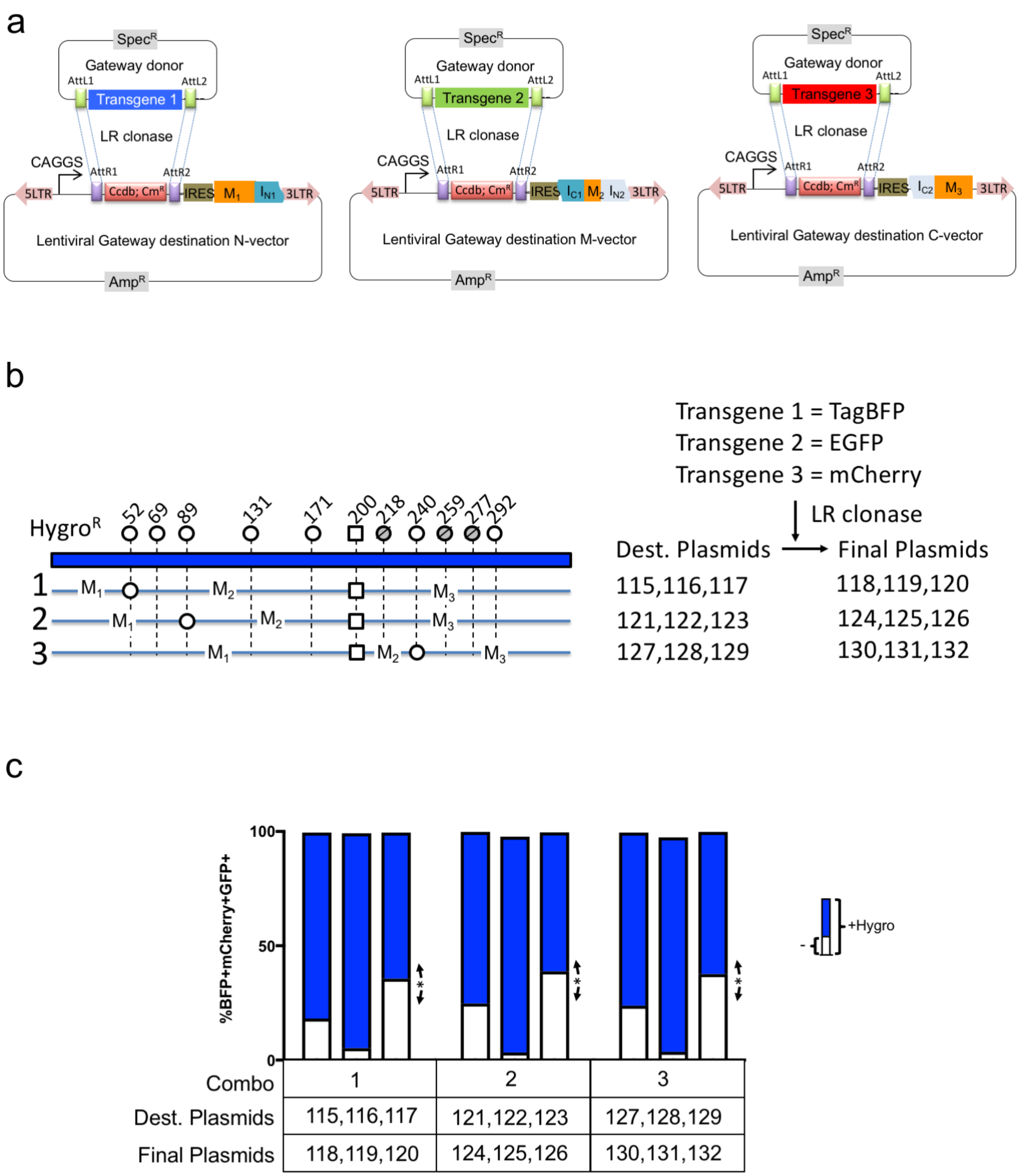

Supplementary figure 8. Gateway-compatible lentiviral destination vectors with 3-split Hygro Intres genes. (a) Gateway-compatible lentiviral destination vector with the viral LTRs, CAGGS promoter, Gateway destination cloning that allows LR clonase-mediated recombination of Gateway donor vector carrying transgenes, followed by internal ribosomal entry site (IRES) that allows polycistronic expression of the each of the three 3-split Hygromycin markertrons. (b) TagBFP (as transgene 1) and EGFP (as transgene 2) and mCherry (as transgene 3) were cloned into the 3-split Intres plasmids by Gateway recombination and delivered to cells by lentiviral transduction, followed by antibiotic selection and flow cytometry analysis. (c) Column plot shows the percentage of BFP+GFP+mCherry+ triple-positive cells in the Hygromycin selected

(white+blue columns) versus their corresponding non-selective cultures (white portion of each column). Vertical asterisks indicate statistical significance by paired two-sided t-test on the percentages of triple-positive cells in the selected cultures vs non-selected cultures within each transfection group (n.s., non-significant; *$p<0.05$, **$p<0.01$,***$p<0.001$).

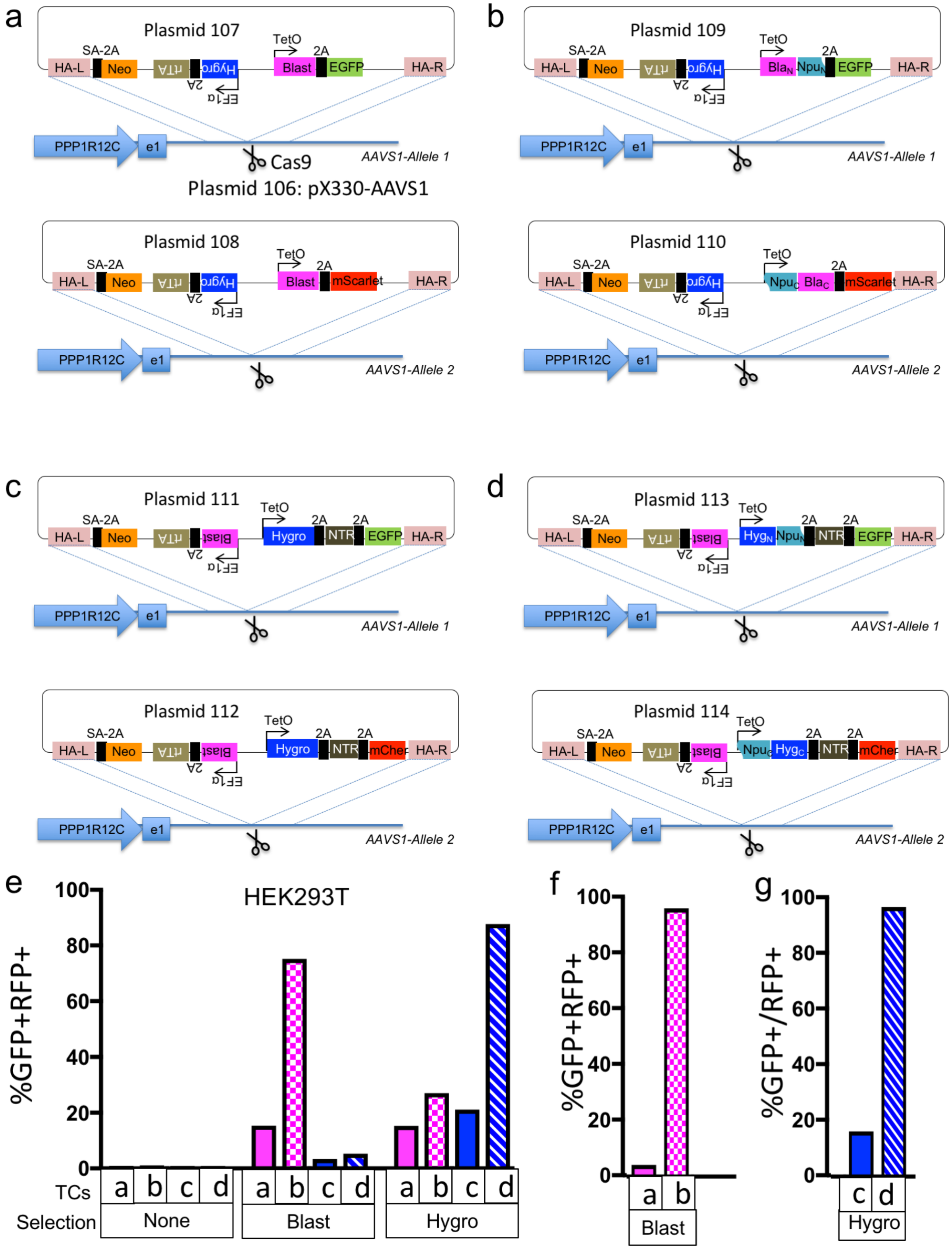

Supplementary figure 9. Intres markers allow enrichment of biallelic targeted cells from CRISPR/Cas-mediated knock-in experiments. Targeting construct pairs containing homology arms for AAVS1 safe harbor locus were designed to contain full length (FL) non-split or split Intres markers and tested for the ability to enrich for biallelic targeted cells via antibiotic selection. (a) Plasmids 107 and 108 contains FL Neomycin (Neo) resistance gene driven by endogenous PPP1R12C

promoter at the AAVS1 locus, FL Hygromycin (Hygro) gene and rtTA Dox-responsive transactivator driven by an EF1a promoter, as well as FL Blasticidin (Blast) expressed as well as EGFP (plasmid 107) and mScarlet (plasmid 108) from a dox-inducible TetO promoter. Plasmid 106 contains Cas9 and an sgRNA targeting the AAVS locus. 2A: self-cleaving 2A peptides. Plasmids 106, 107 and 108 were co-transfected into HEK293T cells, split, and passaged in dox-containing Hygromycin, Blasticidin or non-selective media for two weeks, and analyzed by flow cytometry to assay efficiency of biallelic targeting. (b) Plasmids 109 and 110 contain similar structure as Plasmids 107 and 108, but have split Blast Intres instead of the FL Blast. (c) Plasmids 111 and 112 contain an EF1a-driven FL Blast and TetO-driven FL Hygro, nitroreductase (NTR), fluorescent protein (EGFP or mCherry) separated by 2A peptides. (d) Plasmids 113 and 114 are similar to Plasmids 111 and 112 but with Hygro Intres instead of FL Hygro. (e) Flow cytometry analysis of cells transfected with Plasmid 106 (Cas9+AAVS-sgRNA) and the indicated targeting construct pairs, two weeks after culturing in dox-containing non-selective media (Selection: None), Blasticidin selection media (Blast) and Hygromycin selection media (Hygro). (f) Flow cytometry analysis of cells transfected with Plasmid 106 (Cas9+AAVS-sgRNA) and the indicated targeting construct pairs, four weeks after culturing in dox-containing non-selective media (Selection: None) or Blasticidin selection media (Blast) (g) Flow cytometry analysis of cells transfected with Plasmid 106 (Cas9+AAVS-sgRNA) and the indicated targeting construct pairs, four weeks after culturing in dox-containing non-selective media (Selection: None) or Hygromycin selection media (Hygro).

a

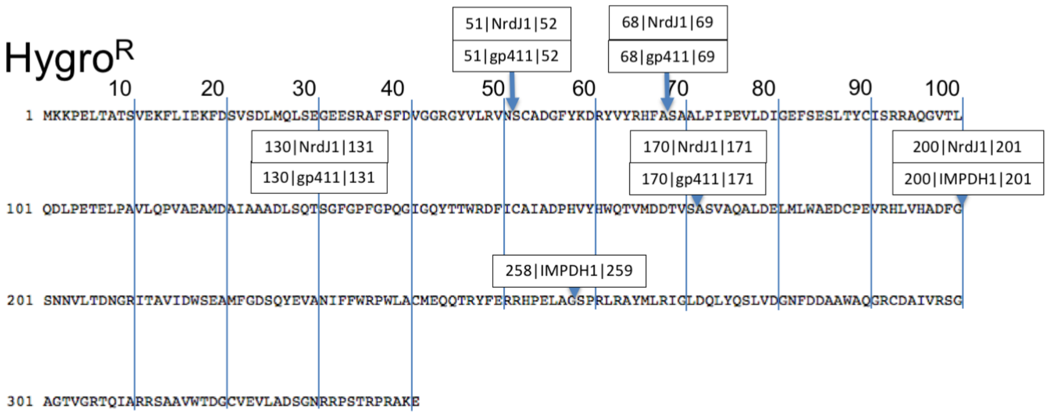

b

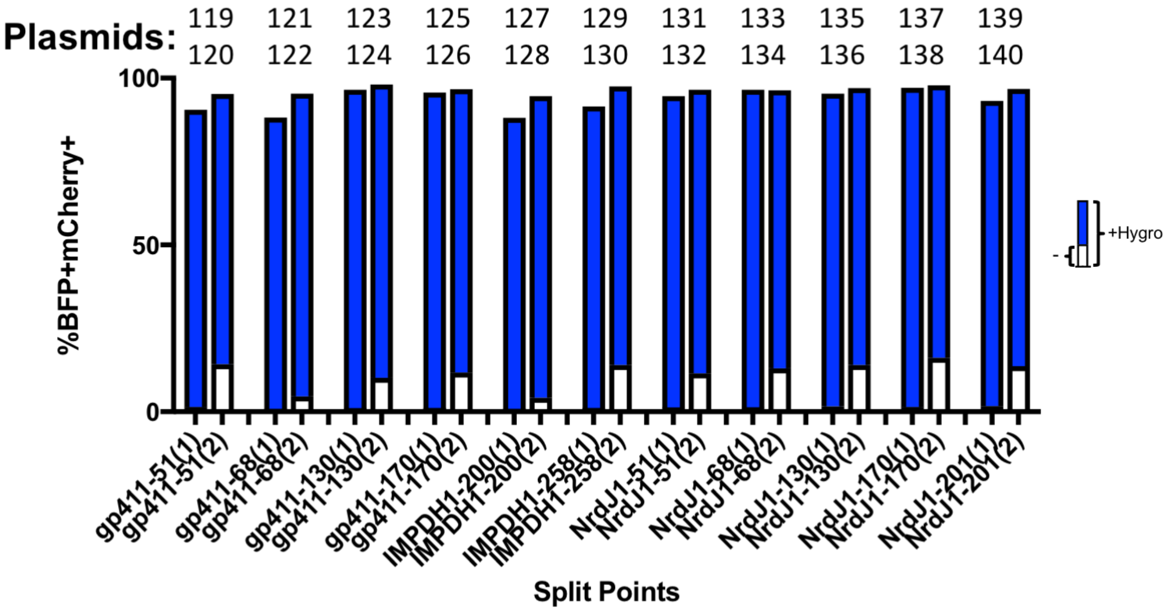

Supplementary figure 10. Additional 2-split Hygro Intres markers. (a) Schematic showing split points for the Hygromycin resistance (Hygro^R) protein. Amino acid sequence of Hygro^R is presented with clouds labeling the split points and inteins. (b) Using assays similar to those shown in Fig 2a, these additional Hygro Intres were tested for double transgenic selection of BFP and mCherry transgenes. Column plot shows percentages of BFP+/mCherry+ double positive cells in non-selective (white portion) and selective cultures (total column height=white+blue) of cells transduced with lentiviruses containing the indicated Intres. Adjacent columns show results from duplicate experiments.

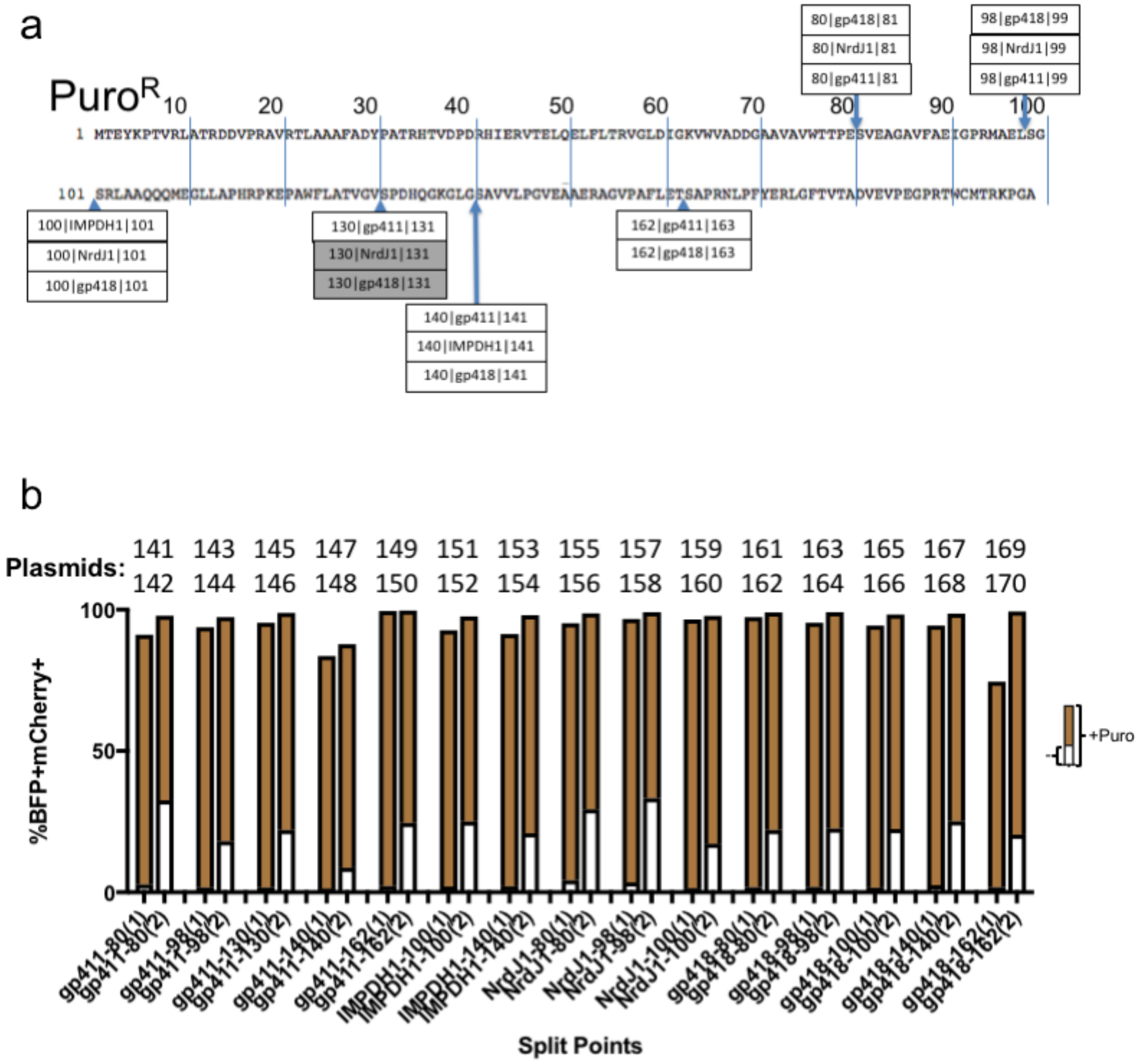

Supplementary figure 11. Additional 2-split Puromycin Intres genes. (a) Schematic showing split points for the Puromycin resistance (Puro^R) protein. Amino acid sequence of Puro^R is presented with clouds labeling the split points and inteins. (b) Using assays similar to those shown in Fig 2a, these additional Puro Intres were tested for double transgenic selection of BFP and mCherry transgenes. Column plot shows percentages of BFP+/mCherry+ double positive cells in non-selective (white portion) and selective cultures (total column height=white+brown) of cells transduced with lentiviruses containing the indicated Intres. Adjacent columns show results from duplicate experiments.

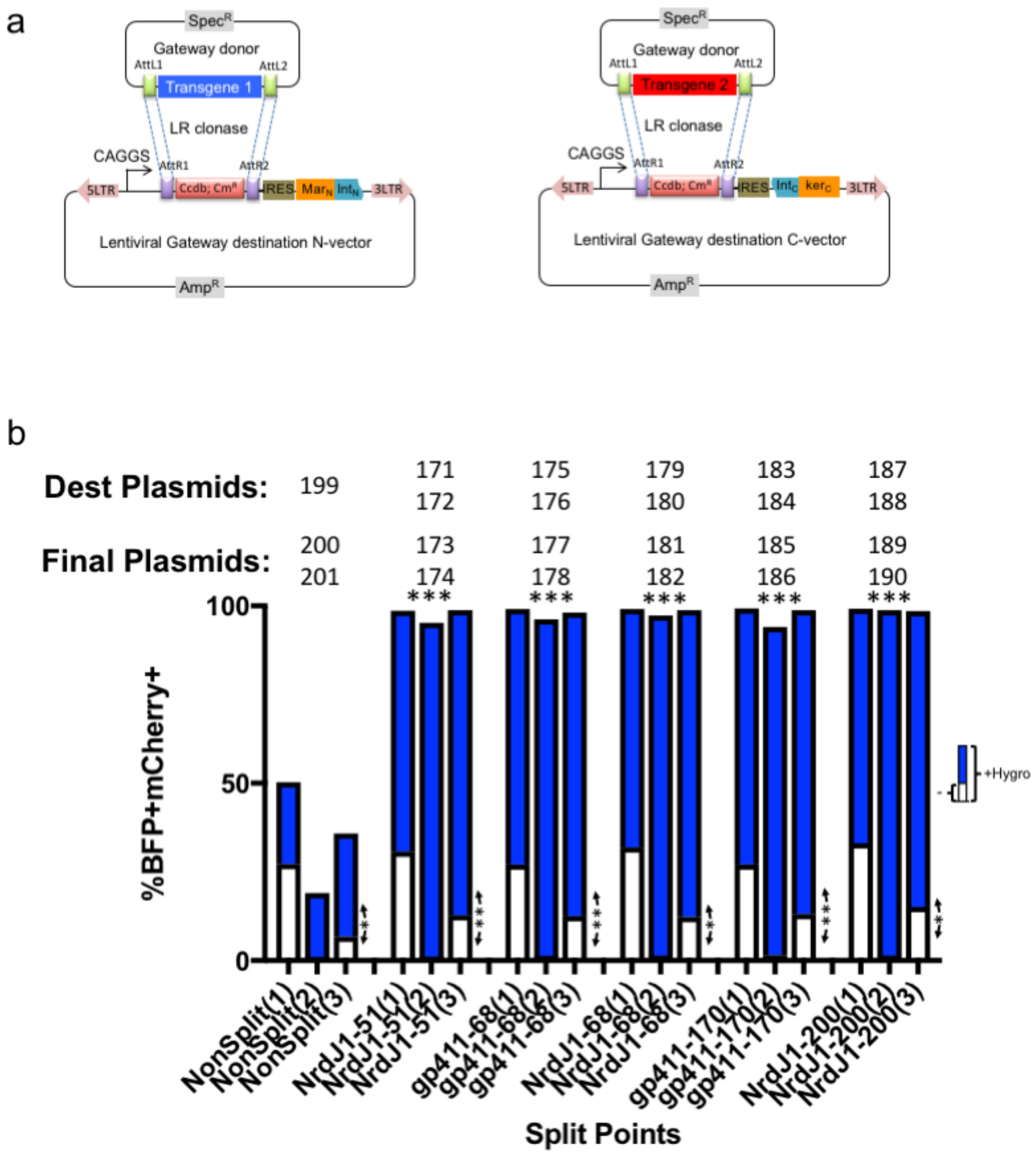

Supplementary figure 12. Additional Gateway-compatible lentiviral destination vectors with 2-split Hygro Intres markers. (a) Gateway-compatible lentiviral destination vector kits for each split Intres marker consists of an N-vector and C-vector. N-vector contains the viral LTRs, CAGGS promoter, gateway destination cloning cassette that allows LR clonase-mediated recombination of Gateway donor vector carrying transgenes, followed by internal ribosomal entry site (IRES) that allows polycistronic expression of the N-markertron. Similarly, C-vector contains the C-markertron and allows recombination of another transgene. (b) TagBFP (as transgene 1) and mCherry (as transgene 2) were cloned into the 2-split Intres plasmids by Gateway recombination and delivered to cells by lentiviral transduction, followed by antibiotic selection and flow cytometry analysis. Column plot shows the percentage of BFP+mCherry+ double-positive cells of non-

selective cultures (white portion) and selective cultures (total column height=white+blue) of cells transduced with lentiviruses containing the indicated non-split Hygro^R or split Hygro Intres markers. Adjacent columns show results from triplicate experiments. Horizontal asterisks indicate statistical significance by one-way ANOVA test on the percentages of double-positive cells in the selected cultures of the specific split marker vs those in the non-split marker (n.s., non-significant; *p<0.05, **p<0.01,***p<0.001). Vertical asterisks indicate statistical significance by paired two-sided t-test on the percentages of double-positive cells in the selected cultures vs non-selected cultures within each transfection group (n.s., non-significant; *p<0.05, **p<0.01,***p<0.001).

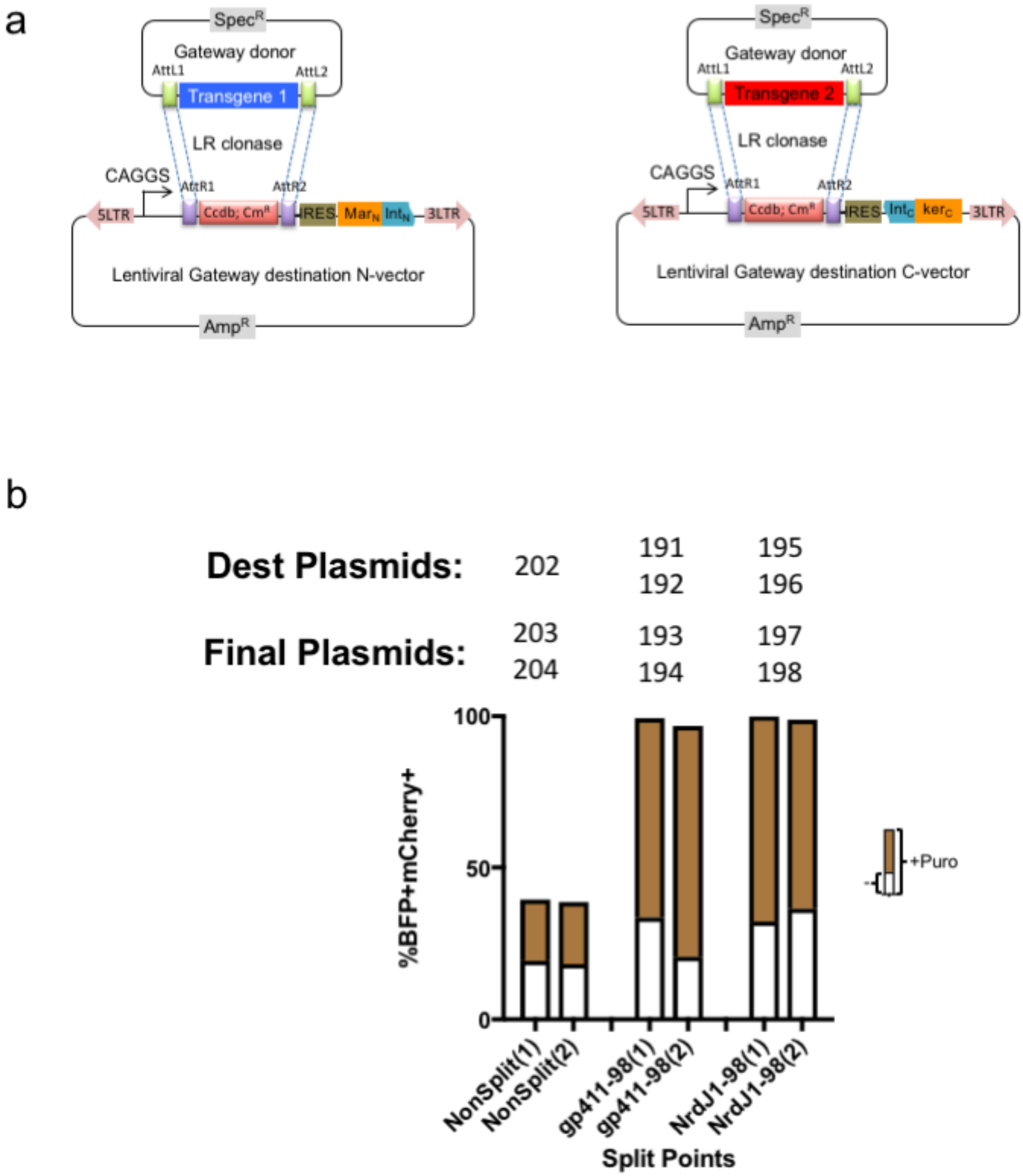

Supplementary figure 13. Additional Gateway-compatible lentiviral destination vectors with 2-split Puro Intres markers. (a) Gateway-compatible lentiviral destination vector kits for each split Intres marker consists of an N-vector and C-vector. N-vector contains the viral LTRs, CAGGS promoter, gateway destination cloning cassette that allows LR clonase-mediated recombination of Gateway donor vector carrying transgenes, followed by internal ribosomal entry site (IRES) that allows polycistronic expression of the N-markertron. Similarly, C-vector contains the C-markertron and allows recombination of another transgene. (b) TagBFP (as transgene 1) and mCherry (as transgene 2) were cloned into the 2-split Intres plasmids by Gateway recombination and delivered to cells by lentiviral transduction, followed by antibiotic

selection and flow cytometry analysis. Column plot shows the percentages of BFP+mCherry+ double-positive cells of non-selective cultures (white portion) and selective cultures (total column height=white+brown) of cells transduced with lentiviruses containing the indicated non-split $Puro^R$ or split Puro Intres markers.

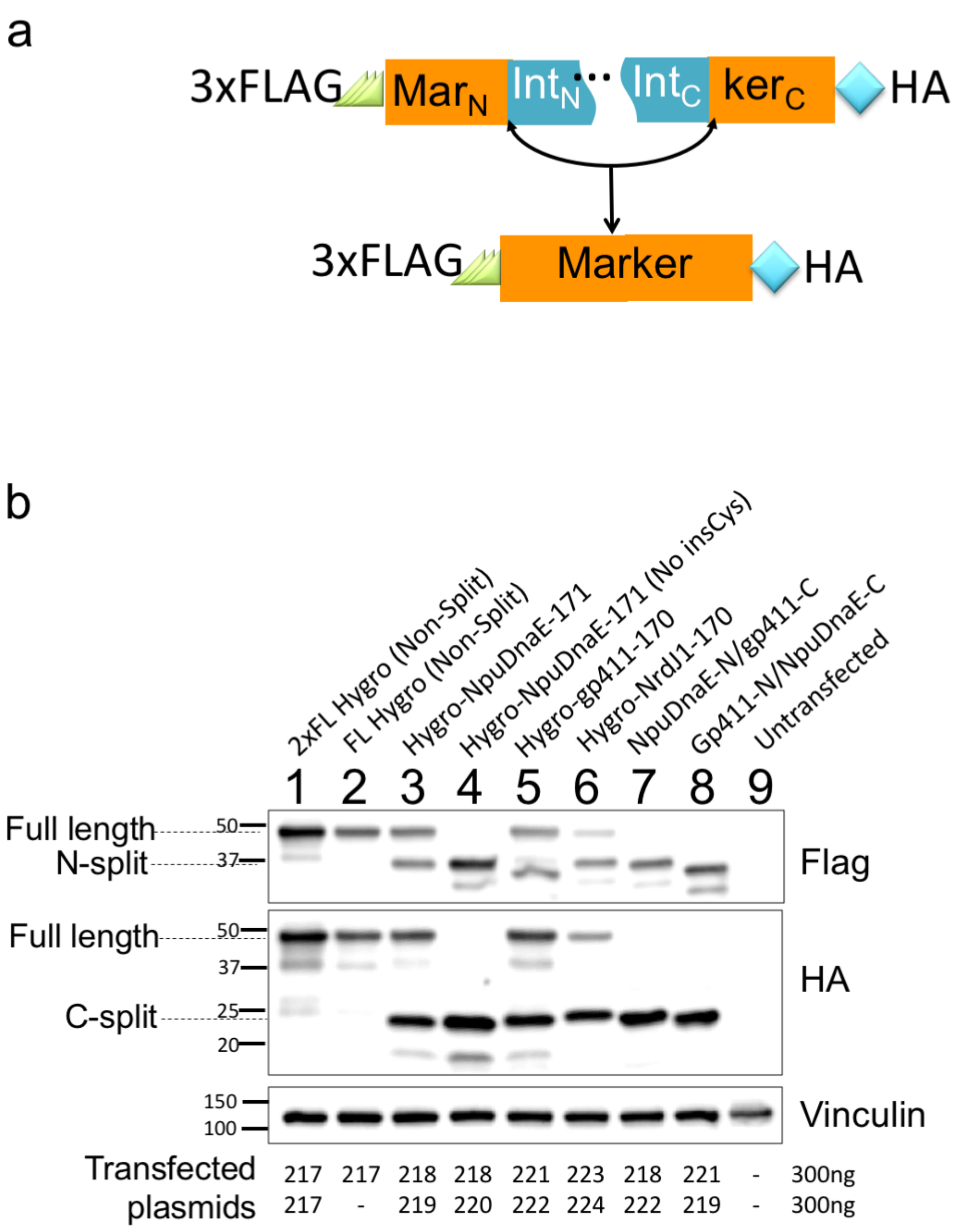

Supplementary figure 14. Western blot analysis of protein trans-splicing of Hygromycin markertrons. (a) A N-markertron is N-terminally tagged with 3xFLAG epitope while a C-marketron is C-terminally tagged with HA epitope. Analysis of trans-splicing is conducted by western blot analysis using anti-FLAG or anti-HA antibodies. (b) Western blot photograph of lysates from HEK293T cells transfected with the indicated N- and C-markertrons or from untransfected cells, using the

indicated antibodies (on the right). The bands corresponding to the N-split, C-split or reconstituted marker proteins are indicated on the left. Vinculin serves as equal-loading control.

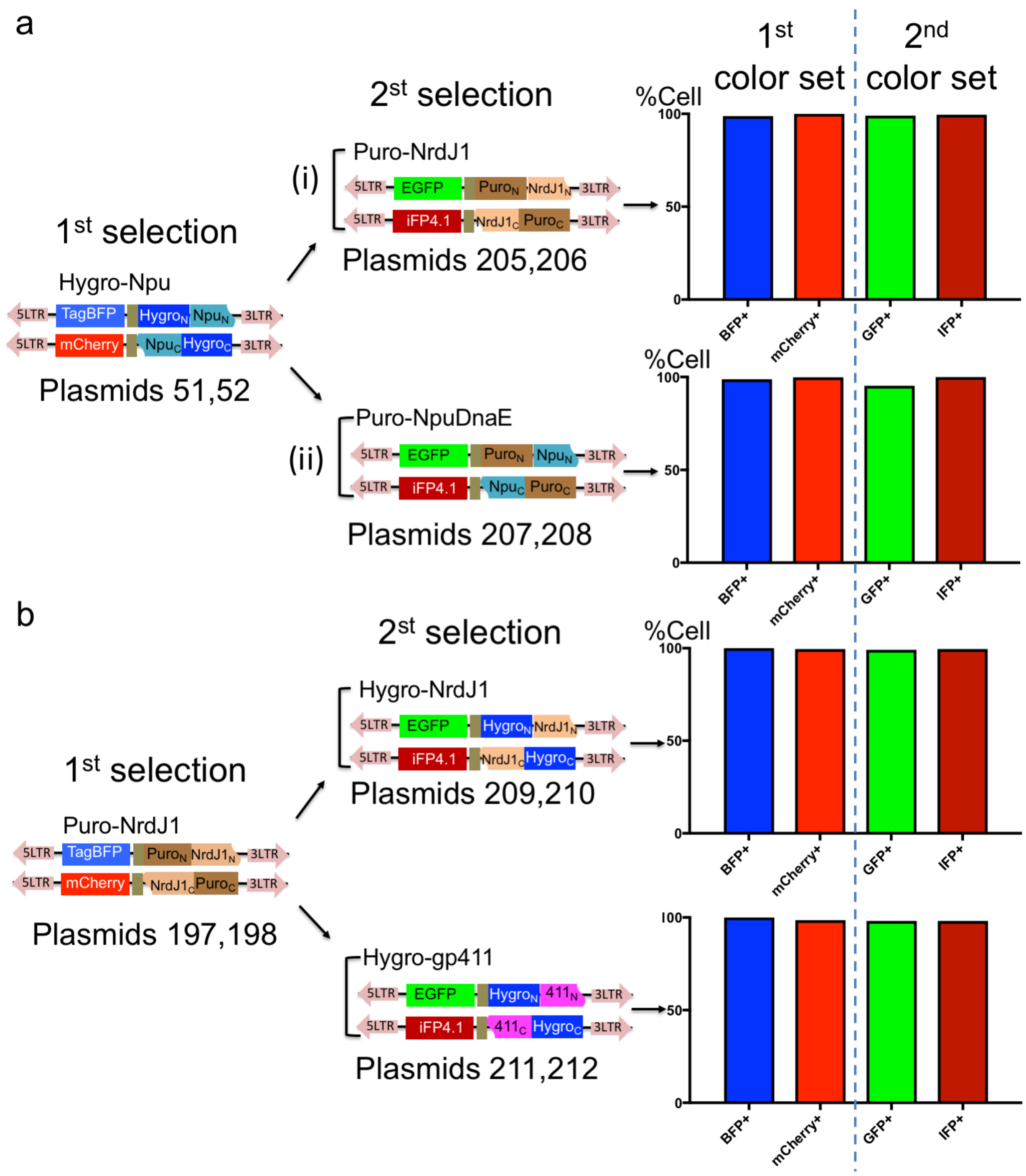

Supplementary figure 15. Two-round sequential transduction/selection using two sets of Intres markers to engineer cells with four transgenes. (a) U2OS cells were first transduced with two lentiviruses carrying TagBFP or mCherry, and Hygro-NpuDnaE Intres, selected with Hygromycin, then further transduced with two lentiviruses carrying EGFP or iFP4.1 and (i) Puro-NrdJ1 or (ii) Puro-NpuDnaE Intres markers, selected with Puromycin, then analyzed for fluorescence from TagBFP, mCherry, EGFP and iFP4.1 by flow cytometry. Column plots show the percentage of cells with the indicated fluorescence in the selected cultures. (b) U2OS cells were first transduced with two lentiviruses carrying TagBFP or mCherry, and Puro-NrdJ1 Intres, selected with Puromycin, then further transduced with two lentiviruses carrying EGFP or iFP4.1 and (i)

Hygro-NrdJ1 or (ii) Hygro-gp411 Intres markers, selected with Hygromycin, then analyzed for fluorescence from TagBFP, mCherry, EGFP and iFP4.1 by flow cytometry. Column plots show the percentage of cells with the indicated fluorescence in the selected cultures.

a

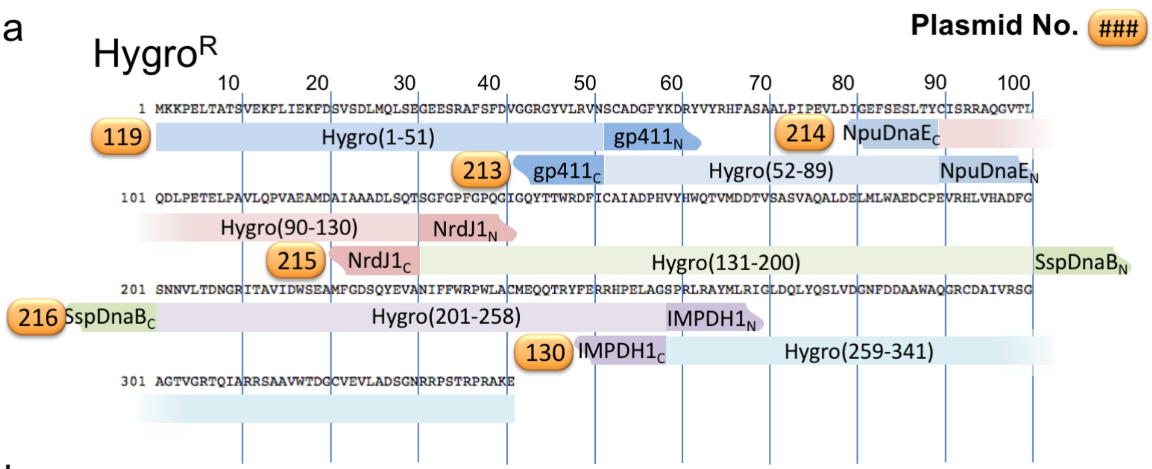

b

Plasmid	Sample 1	Sample 2	Sample 3	Sample 4	Sample 5	Sample 6	Sample 7
119	+	-	+	+	+	+	+
213	+	+	-	+	+	+	+
214	+	+	+	-	+	+	+
215	+	+	+	+	-	+	+
216	+	+	+	+	+	-	+
130	+	+	+	+	+	+	-

c

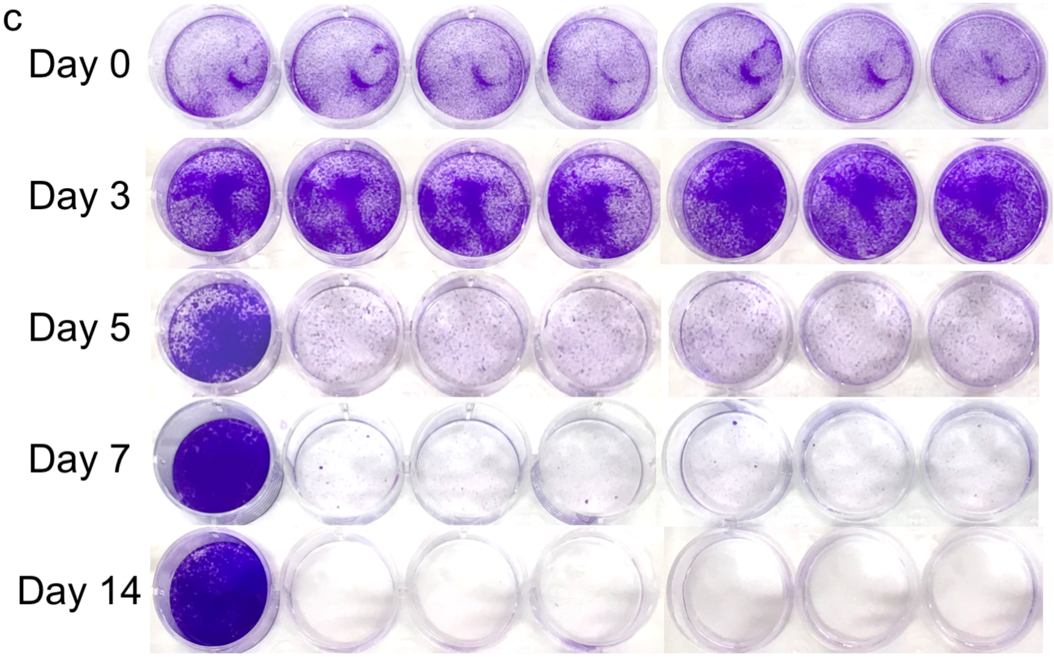

Supplementary figure 16. A 6-split Hygromycin Intres. (a) Schematic showing the amino acid sequence of Hygro^R, the span of each markertron fragment and the fused inteins. (b) U2OS cells were transduced with the complete set or leave-one-out set of lentiviruses carrying the markertrons of the 6-split Hygromycin Intres, as indicated in the table (+: with, -: without), and subjected to Hygromycin selection. Transduction/selection experiments were repeated three times. (c) Representative micrographs of the corresponding cultures stained with Crystal Violet on the indicated days after selection.

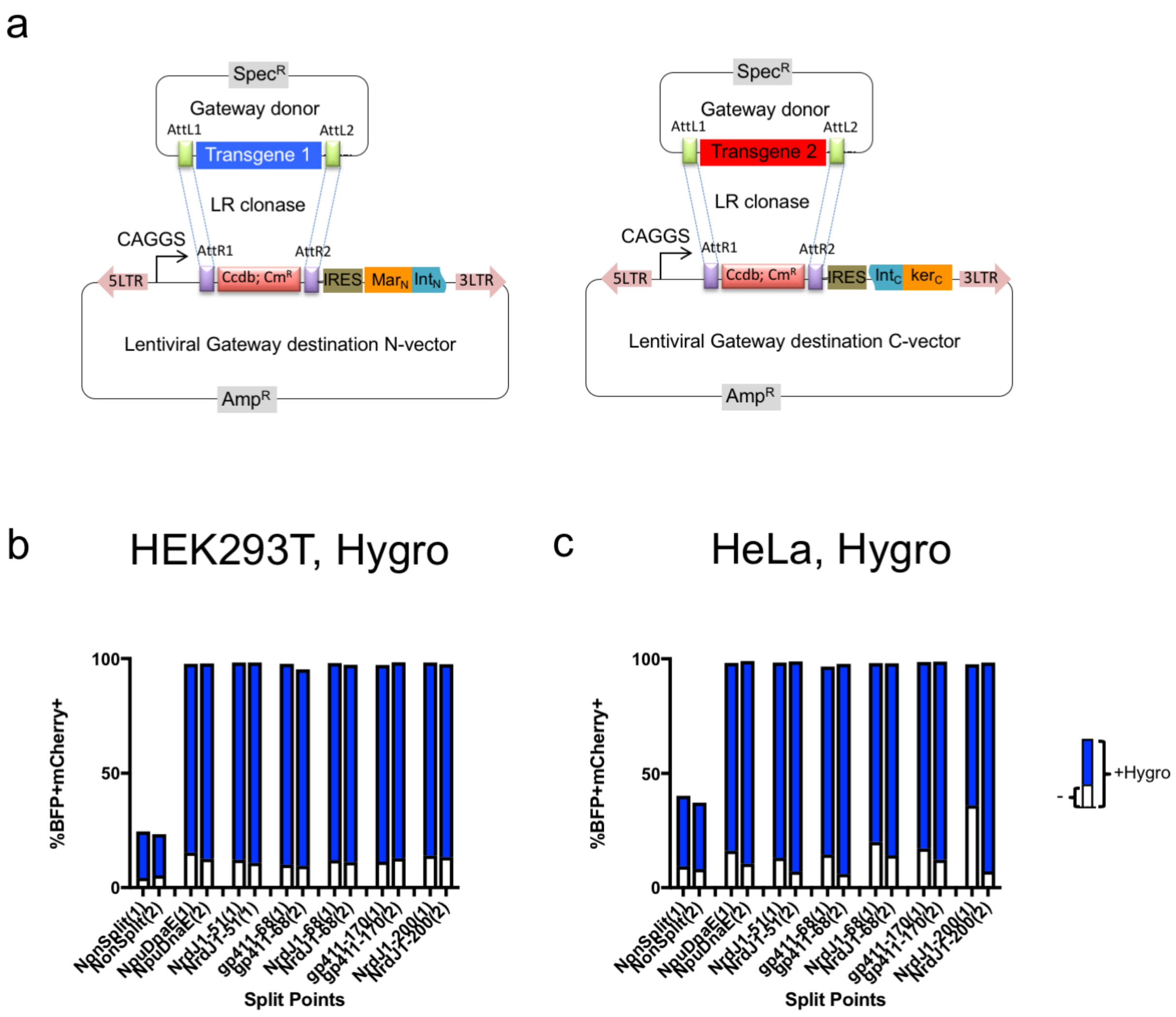

Supplementary figure 17. Application of Gateway-compatible Hygromycin Intres lentiviral vectors in HEK293T and HeLa cells. (a) TagBFP (as transgene 1) and mCherry (as transgene 2) were cloned into the 2-split Hygromycin Intres plasmids by Gateway recombination and delivered to cells by lentiviral transduction, followed by antibiotic selection and flow cytometry analysis. (b) Column plot showing percentages of BFP+mCherry+ double-positive cells from non-selective cultures (white portion) and selective cultures (total column height=white+blue) of HEK293T cells transduced with lentiviruses containing the indicated non-split Hygro or Hygro Intres markers. (c) Column plot showing percentage of BFP+mCherry+ double positive cells from non-selective cultures (white portion) and selective cultures (total column height=white+blue) of HeLa cells transduced with lentiviruses containing the indicated non-split Hygro or Hygro Intres markers.

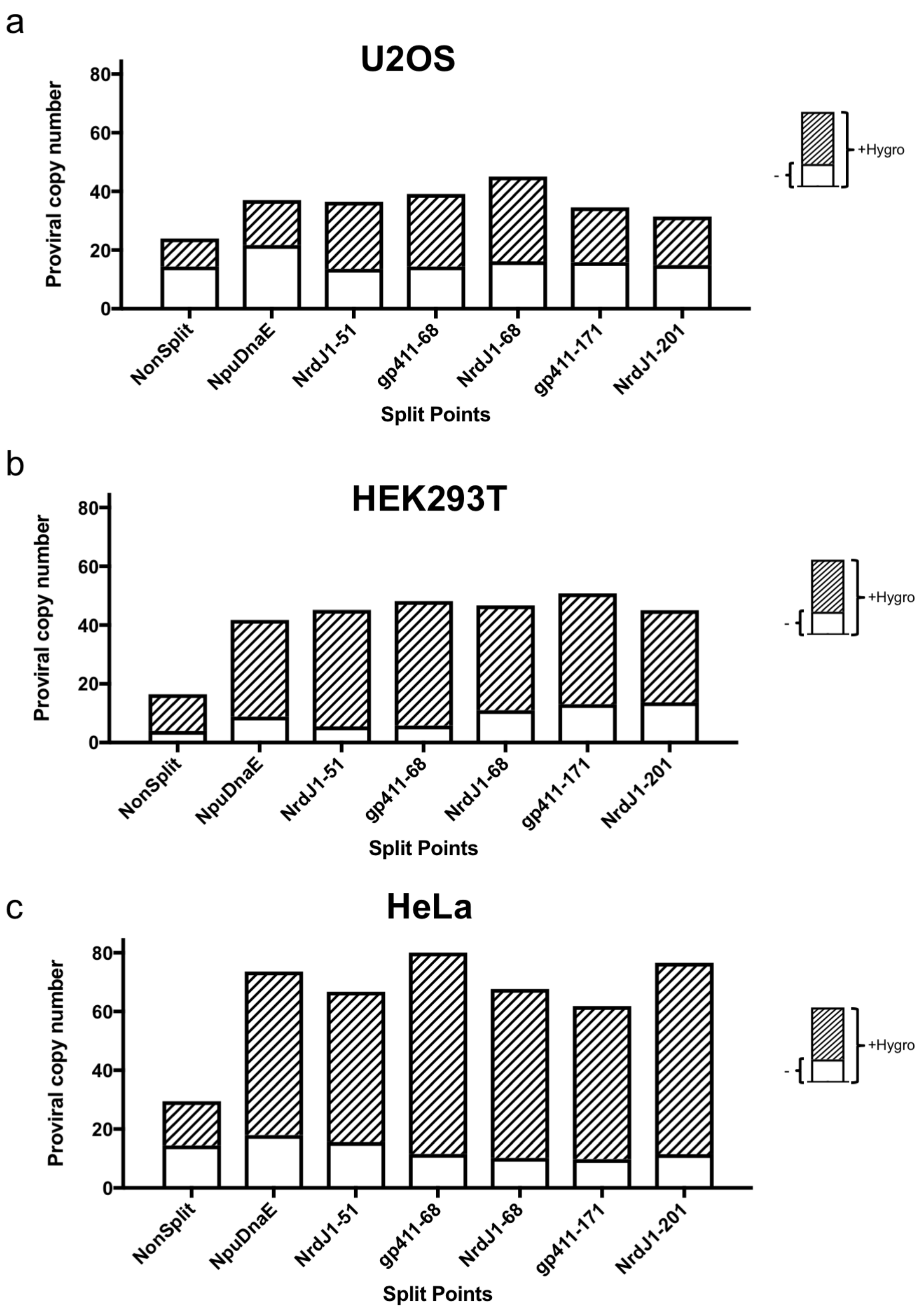

Supplementary figure 18. Proviral copy number analysis of U2OS, HEK293T, HeLa cells transduced with lentiviral vectors containing the indicated non-split Hygro^R or split Hygro Intres markers. (a) Column plot shows proviral copy number of non-selective (white portion) and selective (total column height=white+patterned) cultures of U2OS cells transduced with lentiviruses containing the indicated markers. (b) Column plot shows proviral copy number of non-selective (white portion) and selective (total column height=white+patterned) cultures of HEK293T cells transduced with lentiviruses containing the indicated markers. (c) Column plot shows proviral copy number of non-selective (white portion) and selective (total column height=white+patterned) cultures of HeLa cells transduced with lentiviruses containing the indicated markers.

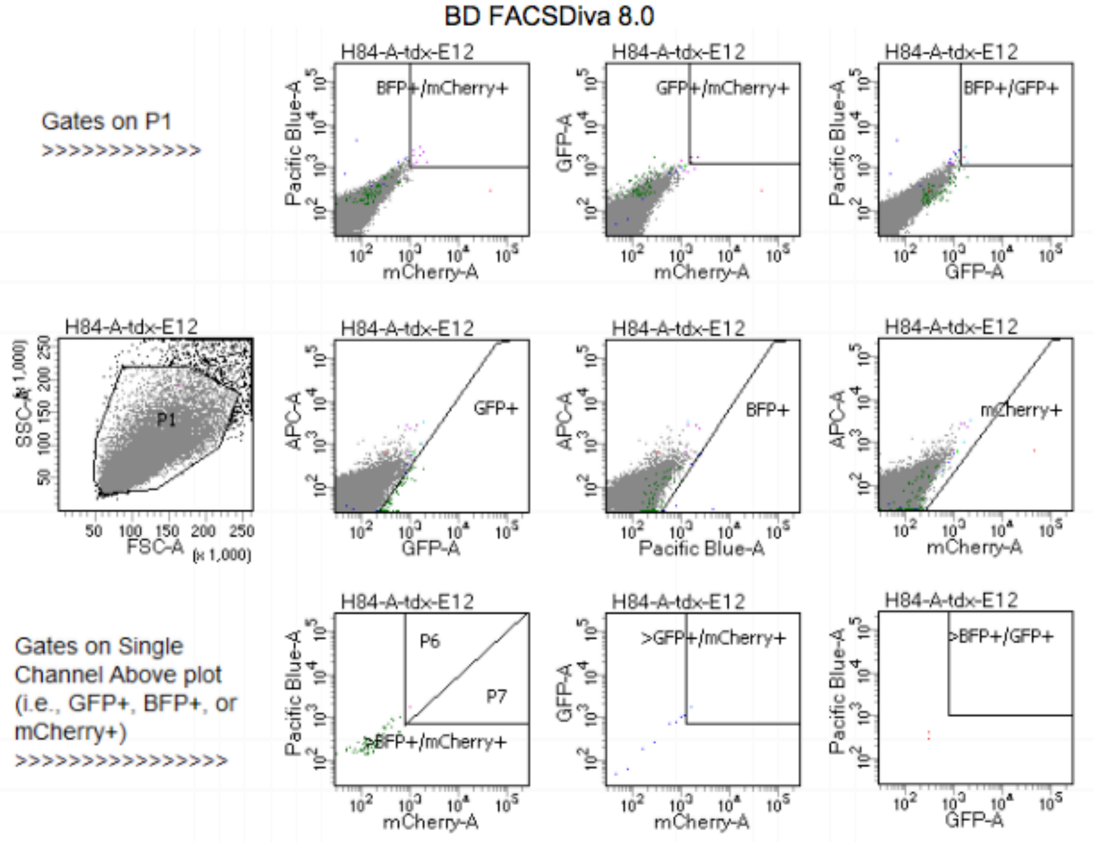

Well: E12

Population	#Events	%Parent	%Total
All Events	50,000	####	100.0
P1	48,478	97.0	97.0
GFP+	72	0.1	0.1
>BFP+/mCherry	1	1.4	0.0
P6	1	1.4	0.0
P7	0	0.0	0.0
mCherry+	2	0.0	0.0
>BFP+/GFP+	0	0.0	0.0
BFP+	9	0.0	0.0
>GFP+/mCherry	1	11.1	0.0
BFP+/mCherry+	8	0.0	0.0
GFP+2	3	37.5	0.0
GFP+/mCherry+	3	0.0	0.0
BFP+/GFP+	3	0.0	0.0

Supplementary figure 19. Flow cytometry gating strategy. Flow cytometry gates were defined by running an untransfected sample and setting the boundaries based on the scatters of these non-fluorescent cells in each channel.

Supplementary Table 1

Plasmid #	pAC#	Plasmid Name	Markertron/Marker	Plasmid Info
3	pAC10003	pLX-Hygro(1-89)-NpuDnaE(N)-IRES-TagBFP2	Hygro(1-89)-NpuDnaE(N)	http://vectors.intr.es/?k=pAC10003
4	pAC10004	pLX-NpuDnaE(C)-Hygro(90-341)-IRES-mCherry	NpuDnaE(C)-Hygro(90-341)	http://vectors.intr.es/?k=pAC10004
5	pAC10005	pLX-Hygro(1-200)-SspDnaB(N)-IRES-TagBFP2	Hygro(1-200)-SspDnaB(N)	http://vectors.intr.es/?k=pAC10005
6	pAC10006	pLX-SspDnaB(C)-Hygro(201-341)-IRES-mCherry	SspDnaB(C)-Hygro(201-341)	http://vectors.intr.es/?k=pAC10006
7	pAC10007	pLX-Hygro(1-52)-NpuDnaE(N)-IRES-TagBFP2	Hygro(1-52)-NpuDnaE(N)	http://vectors.intr.es/?k=pAC10007
8	pAC10008	pLX-NpuDnaE(C)-Hygro(53-341)-IRES-mCherry	NpuDnaE(C)-Hygro(53-341)	http://vectors.intr.es/?k=pAC10008
9	pAC10009	pLX-Hygro(1-240)-NpuDnaE(N)-IRES-TagBFP2	Hygro(1-240)-NpuDnaE(N)	http://vectors.intr.es/?k=pAC10009
10	pAC10010	pLX-NpuDnaE(C)-Hygro(241-341)-IRES-mCherry	NpuDnaE(C)-Hygro(241-341)	http://vectors.intr.es/?k=pAC10010
11	pAC10011	pLX-Hygro(1-292)-NpuDnaE(N)-IRES-TagBFP2	Hygro(1-292)-NpuDnaE(N)	http://vectors.intr.es/?k=pAC10011
12	pAC10012	pLX-NpuDnaE(C)-Hygro(293-341)-IRES-mCherry	NpuDnaE(C)-Hygro(293-341)	http://vectors.intr.es/?k=pAC10012
13	pAC10013	pLX-Blast(1-102)-NpuDnaE(N)-IRES-TagBFP2	Blast(1-102)-NpuDnaE(N)	http://vectors.intr.es/?k=pAC10013
14	pAC10014	pLX-NpuDnaE(C)-Blast(103-140)-IRES-mCherry	NpuDnaE(C)-Blast(103-140)	http://vectors.intr.es/?k=pAC10014
17	pAC10017	pLX-Puro(1-119)-NpuDnaE(N)-IRES-TagBFP2	Puro(1-119)-NpuDnaE(N)	http://vectors.intr.es/?k=pAC10017
18	pAC10018	pLX-NpuDnaE(C)-Puro(insCys;120-199)-IRES-mCherry	NpuDnaE(C)-Puro(insCys;120-199)	http://vectors.intr.es/?k=pAC10018
19	pAC10019	pLX-Puro(1-100)-SspDnaB(N-S0)-IRES-TagBFP2	Puro(1-100)-SspDnaB(N-S0)	http://vectors.intr.es/?k=pAC10019
20	pAC10020	pLX-SspDnaB(C-S0)-Puro(101-199)-IRES-mCherry	SspDnaB(C-S0)-Puro(101-199)	http://vectors.intr.es/?k=pAC10020
21	pAC10021	pLX-Neo(1-133)-NpuDnaE(N)-IRES-TagBFP2	Neo(1-133)-NpuDnaE(N)	http://vectors.intr.es/?k=pAC10021
22	pAC10022	pLX-NpuDnaE(C)-Neo(134-267)-IRES-mCherry	NpuDnaE(C)-Neo(134-267)	http://vectors.intr.es/?k=pAC10022
23	pAC10023	pLX-Neo(1-194)-NpuDnaE(N)-IRES-TagBFP2	Neo(1-194)-NpuDnaE(N)	http://vectors.intr.es/?k=pAC10023
24	pAC10024	pLX-NpuDnaE(C)-Neo(195-267)-IRES-mCherry	NpuDnaE(C)-Neo(195-267)	http://vectors.intr.es/?k=pAC10024
25	pAC10025	pLX-NpuDnaE(C)_Hygro(53-89)-NpuDnaE(N)-IRES-GFP	NpuDnaE(C)_Hygro(53-89)-NpuDnaE(N)	http://vectors.intr.es/?k=pAC10025
26	pAC10026	pLX-NpuDnaE(C)_Hygro(53-239)-NpuDnaE(N)-IRES-GFP	NpuDnaE(C)_Hygro(53-239)-NpuDnaE(N)	http://vectors.intr.es/?k=pAC10026
27	pAC10027	pCR8-BsaI->ccdbCam<-BsaI-NpuDnaE(N)		http://vectors.intr.es/?k=pAC10027
28	pAC10028	pCR8-NpuDnaE(C)_BsaI->ccdbCam<-BsaI		http://vectors.intr.es/?k=pAC10028

31	pAC10031	pCR8-BsaI->ccdbCam<-BsaI-SspDnaB(N-S0)		http://vectors.intr.es/?k=pAC10031
32	pAC10032	pCR8-SspDnaB(C-S0)_BsaI->ccdbCam<-BsaI		http://vectors.intr.es/?k=pAC10032
33	pAC10033	pLX-mScarlet(1-46)-NpuDnaE(N)_LZA-IRES-TagBFP2	mScarlet(1-46)-NpuDnaE(N)_LZA	http://vectors.intr.es/?k=pAC10033
34	pAC10034	pLX-LZB_NpuDnaE(C)-mScarlet(insCys;47-232)-IRES-TagBFP2	LZB_NpuDnaE(C)-mScarlet(insCys;47-232)	http://vectors.intr.es/?k=pAC10034
35	pAC10035	pLX-mScarlet(1-48)-NpuDnaE(N)_LZA-IRES-TagBFP2	mScarlet(1-48)-NpuDnaE(N)_LZA	http://vectors.intr.es/?k=pAC10035
36	pAC10036	pLX-LZB_NpuDnaE(C)-mScarlet(insCys;49-232)-IRES-GFP	LZB_NpuDnaE(C)-mScarlet(insCys;49-232)	http://vectors.intr.es/?k=pAC10036
37	pAC10037	pLX-mScarlet(1-51)-NpuDnaE(N)_LZA -IRES-TagBFP2	mScarlet(1-51)-NpuDnaE(N)_LZA	http://vectors.intr.es/?k=pAC10037
38	pAC10038	pLX-LZB_NpuDnaE(C)-mScarlet(insCys;52-232)-IRES-GFP	LZB_NpuDnaE(C)-mScarlet(insCys;52-232)	http://vectors.intr.es/?k=pAC10038
39	pAC10039	pLX-mScarlet(1-75)-NpuDnaE(N)_LZA-IRES-TagBFP2	mScarlet(1-75)-NpuDnaE(N)_LZA	http://vectors.intr.es/?k=pAC10039
40	pAC10040	pLX-LZB_NpuDnaE(C)-mScarlet(insCys;76-232)-IRES-GFP	LZB_NpuDnaE(C)-mScarlet(insCys;76-232)	http://vectors.intr.es/?k=pAC10040
41	pAC10041	pLX-mScarlet(1-122)-NpuDnaE(N)_LZA-IRES-TagBFP2	mScarlet(1-122)-NpuDnaE(N)_LZA	http://vectors.intr.es/?k=pAC10041
42	pAC10042	pLX-LZB_NpuDnaE(C)-mScarlet(insCys;123-232)-IRES-GFP	LZB_NpuDnaE(C)-mScarlet(insCys;123-232)	http://vectors.intr.es/?k=pAC10042
43	pAC10043	pLX-mScarlet(1-140)-NpuDnaE(N)_LZA-IRES-TagBFP2	mScarlet(1-140)-NpuDnaE(N)_LZA	http://vectors.intr.es/?k=pAC10043
44	pAC10044	pLX-LZB_NpuDnaE(C)-mScarlet(insCys;141-232)-IRES-GFP	LZB_NpuDnaE(C)-mScarlet(insCys;141-232)	http://vectors.intr.es/?k=pAC10044
45	pAC10045	pLX-mScarlet(1-163)-NpuDnaE(N)_LZA-IRES-TagBFP2	mScarlet(1-163)-NpuDnaE(N)_LZA	http://vectors.intr.es/?k=pAC10045
46	pAC10046	pLX-LZB_NpuDnaE(C)-mScarlet(insCys;164-232)-IRES-GFP	LZB_NpuDnaE(C)-mScarlet(insCys;164-232)	http://vectors.intr.es/?k=pAC10046
47	pAC10047	pCR8-TagBFP2	TagBFP2	http://vectors.intr.es/?k=pAC10047
48	pAC10048	pCR8-mCherry	mCherry	http://vectors.intr.es/?k=pAC10048
49	pAC10049	pLX-DEST-IRES-Hygro(1-89)-NpuDnaE(N)	Hygro(1-89)-NpuDnaE(N)	http://vectors.intr.es/?k=pAC10049
50	pAC10050	pLX-DEST-IRES-NpuDnaE(C)-Hygro(90-341)	NpuDnaE(C)-Hygro(90-341)	http://vectors.intr.es/?k=pAC10050
51	pAC10051	pLX-[TagBFP2]-IRES-Hygro(1-89)-NpuDnaE(N)	Hygro(1-89)-NpuDnaE(N)	http://vectors.intr.es/?k=pAC10051
52	pAC10052	pLX-[mCherry]-IRES-NpuDnaE(C)-Hygro(90-341)	NpuDnaE(C)-Hygro(90-341)	http://vectors.intr.es/?k=pAC10052
53	pAC10053	pLX-DEST-IRES-Puro(1-119)-NpuDnaE(N)	Puro(1-119)-NpuDnaE(N)	http://vectors.intr.es/?k=pAC10053
54	pAC10054	pLX-DEST-IRES-NpuDnaE(C)-	NpuDnaE(C)-	http://vectors.intr.es/?k=pAC10054

		Puro(120-199)	Puro(insCys;120-199)	
55	pAC10055	pLX-[TagBFP2]-IRES-Puro(1-119)-NpuDnaE(N)	Puro(1-119)-NpuDnaE(N)	http://vectors.intr.es/?k=pAC10055
56	pAC10056	pLX-[mCherry]-IRES-NpuDnaE(C)-Puro(120-199)	NpuDnaE(C)-Puro(insCys;120-199)	http://vectors.intr.es/?k=pAC10056
57	pAC10057	pLX-DEST-IRES-Neo(1-194)-NpuDnaE(N)	Neo(1-194)-NpuDnaE(N)	http://vectors.intr.es/?k=pAC10057
58	pAC10058	pLX-DEST-IRES-NpuDnaE(C)-Neo(195-267)	NpuDnaE(C)-Neo(195-267)	http://vectors.intr.es/?k=pAC10058
59	pAC10059	pLX-[TagBFP2]-IRES-Neo(1-194)-NpuDnaE(N)	Neo(1-194)-NpuDnaE(N)	http://vectors.intr.es/?k=pAC10059
60	pAC10060	pLX-[mCherry]-IRES-NpuDnaE(C)-Neo(195-267)	NpuDnaE(C)-Neo(195-267)	http://vectors.intr.es/?k=pAC10060
64	pAC10064	pLX-Hygro(1-69)-NpuDnaE(N)-IRES-TagBFP2	Hygro(1-69)-NpuDnaE(N)	http://vectors.intr.es/?k=pAC10064
65	pAC10065	pLX-NpuDnaE(C)-Hygro(^C;70-341)-IRES-mCherry	NpuDnaE(C)-Hygro(^C;70-341)	http://vectors.intr.es/?k=pAC10065
66	pAC10066	pLX-Hygro(1-131)-NpuDnaE(N)-IRES-TagBFP2	Hygro(1-131)-NpuDnaE(N)	http://vectors.intr.es/?k=pAC10066
67	pAC10067	pLX-NpuDnaE(C)-Hygro(^C;132-341)-IRES-mCherry	NpuDnaE(C)-Hygro(^C;132-341)	http://vectors.intr.es/?k=pAC10067
68	pAC10068	pLX-Hygro(1-171)-NpuDnaE(N)-IRES-TagBFP2	Hygro(1-171)-NpuDnaE(N)	http://vectors.intr.es/?k=pAC10068
69	pAC10069	pLX-NpuDnaE(C)-Hygro(^C;172-341)-IRES-mCherry	NpuDnaE(C)-Hygro(^C;172-341)	http://vectors.intr.es/?k=pAC10069
70	pAC10070	pLX-Hygro(1-218)-NpuDnaE(N)-IRES-TagBFP2	Hygro(1-218)-NpuDnaE(N)	http://vectors.intr.es/?k=pAC10070
71	pAC10071	pLX-NpuDnaE(C)-Hygro(^C;219-341)-IRES-mCherry	NpuDnaE(C)-Hygro(^C;219-341)	http://vectors.intr.es/?k=pAC10071
72	pAC10072	pLX-Hygro(1-259)-NpuDnaE(N)-IRES-TagBFP2	Hygro(1-259)-NpuDnaE(N)	http://vectors.intr.es/?k=pAC10072
73	pAC10073	pLX-NpuDnaE(C)-Hygro(^C;260-341)-IRES-mCherry	NpuDnaE(C)-Hygro(^C;260-341)	http://vectors.intr.es/?k=pAC10073
74	pAC10074	pLX-Hygro(1-277)-NpuDnaE(N)-IRES-TagBFP2	Hygro(1-277)-NpuDnaE(N)	http://vectors.intr.es/?k=pAC10074
75	pAC10075	pLX-NpuDnaE(C)-Hygro(^C; 278-341)-IRES-mCherry	NpuDnaE(C)-Hygro(^C;278-341)	http://vectors.intr.es/?k=pAC10075
76	pAC10076	pLX-Puro(1-32)-NpuDnaE(N)-IRES-TagBFP2	Puro(1-32)-NpuDnaE(N)	http://vectors.intr.es/?k=pAC10076
77	pAC10077	pLX-NpuDnaE(C)-Puro(^C;33-199)-IRES-mCherry	NpuDnaE(C)-Puro(^C;33-199)	http://vectors.intr.es/?k=pAC10077
78	pAC10078	pLX-Puro(1-84)-NpuDnaE(N)-IRES-TagBFP2	Puro(1-84)-NpuDnaE(N)	http://vectors.intr.es/?k=pAC10078
79	pAC10079	pLX-NpuDnaE(C)-Puro(^C;85-199)-IRES-mCherry	NpuDnaE(C)-Puro(^C;85-199)	http://vectors.intr.es/?k=pAC10079
80	pAC10080	pLX-Puro(1-137)-NpuDnaE(N)-IRES-TagBFP2	Puro(1-137)-NpuDnaE(N)	http://vectors.intr.es/?k=pAC10080
81	pAC10081	pLX-NpuDnaE(C)-Puro(^C;138-199)-IRES-mCherry	NpuDnaE(C)-Puro(^C;138-199)	http://vectors.intr.es/?k=pAC10081
82	pAC10082	pLX-Puro(1-158)-NpuDnaE(N)-IRES-TagBFP2	Puro(1-158)-NpuDnaE(N)	http://vectors.intr.es/?k=pAC10082
83	pAC10083	pLX-NpuDnaE(C)-Puro(^C;159-	NpuDnaE(C)-Puro(^C;159-	http://vectors.intr.es/?k=pAC10083

		199)-IRES-mCherry	199)	
84	pAC10084	pLX-Puro(1-180)-NpuDnaE(N)-IRES-TagBFP2	Puro(1-180)-NpuDnaE(N)	http://vectors.intr.es/?k=pAC10084
85	pAC10085	pLX-NpuDnaE(C)-Puro(^C;181-199)-IRES-mCherry	NpuDnaE(C)-Puro(^C;181-199)	http://vectors.intr.es/?k=pAC10085
86	pAC10086	pLX-Blast(1-58)-NpuDnaE(N)-IRES-TagBFP2	Blast(1-58)-NpuDnaE(N)	http://vectors.intr.es/?k=pAC10086
87	pAC10087	pLX-NpuDnaE(C)-Blast(59-140)-IRES-mCherry	NpuDnaE(C)-Blast(59-140)	http://vectors.intr.es/?k=pAC10087
88	pAC10088	pLX-NpuDnaE(C)-HygroBA-SspDnaB(N-S0)-IRES-EGFP	NpuDnaE(C)-Hygro(53-200)-SspDnaB(N-S0)	http://vectors.intr.es/?k=pAC10088
89	pAC10089	pLX-SspDnaB(C-S0)-Hygro(201-341)-IRES-mCherry	SspDnaB(C-S0)-Hygro(201-341)	http://vectors.intr.es/?k=pAC10089
90	pAC10090	pLX-NpuDnaE(C)-Hygro(90-200)-SspDnaB(N-S0)-IRES-EGFP	NpuDnaE(C)-Hygro(90-200)-SspDnaB(N-S0)	http://vectors.intr.es/?k=pAC10090
91	pAC10091	pLX-Hygro(1-200)-SspDnaB(N-S0)-IRES-TagBFP2	Hygro(1-200)-SspDnaB(N-S0)	http://vectors.intr.es/?k=pAC10091
92	pAC10092	pLX-SspDnaB(C-S0)-Hygro(201-240)-NpuDnaE(N)-IRES-EGFP	SspDnaB(C-S0)-Hygro(201-240)-NpuDnaE(N)	http://vectors.intr.es/?k=pAC10092
93	pAC10093	pLX-SspDnaB(C-S0)-Hygro(201-292)-NpuDnaE(N)-IRES-EGFP	SspDnaB(C-S0)-Hygro(201-292)-NpuDnaE(N)	http://vectors.intr.es/?k=pAC10093
94	pAC10094	pLX-DEST-IRES-TagBFP2	TagBFP2	http://vectors.intr.es/?k=pAC10094
95	pAC10095	pLX-DEST-IRES-EGFP	EGFP	http://vectors.intr.es/?k=pAC10095
96	pAC10096	pLX-DEST-IRES-mCherry	mCherry	http://vectors.intr.es/?k=pAC10096
97	pAC10097	pLX-Hygro-IRES-TagBFP2	Non-split Hygro	http://vectors.intr.es/?k=pAC10097
98	pAC10098	pLX-Hygro-IRES-mCherry	Non-split Hygro	http://vectors.intr.es/?k=pAC10098
99	pAC10099	pLX-Puro-IRES-TagBFP2	Non-split Puro	http://vectors.intr.es/?k=pAC10099
100	pAC10100	pLX-Puro-IRES-mCherry	Non-split Puro	http://vectors.intr.es/?k=pAC10100
101	pAC10101	pLX-Hygro-IRES-EGFP	Non-split Hygro	http://vectors.intr.es/?k=pAC10101
102	pAC10102	pLX-NLS_GFP-IRES-Hygro	Non-split Hygro	http://vectors.intr.es/?k=pAC10102
103	pAC10103	pLX-LifeAct_mCherry-IRES-Hygro	Non-split Hygro	http://vectors.intr.es/?k=pAC10103
104	pAC10104	pLX-NLS_GFP-IRES-Hygro(1-89)-NpuDnaE(N)	Hygro(1-89)-NpuDnaE(N)	http://vectors.intr.es/?k=pAC10104
105	pAC10105	pLX-LifeAct_mScarlet-IRES-NpuDnaE(C)-Hygro(90-341)	NpuDnaE(C)-Hygro(90-341)	http://vectors.intr.es/?k=pAC10105
106	pAC10106	pX330-AAVS1		http://vectors.intr.es/?k=pAC10106
107	pAC10107	pAAVS1-Nst-EF1aHygro2ArtTA3(-)_TetO-Blast-P2A-EGFP	Blast-P2A-EGFP	http://vectors.intr.es/?k=pAC10107
108	pAC10108	pAAVS1-Nst-EF1aHygro2ArtTA3(-)_TetO-Blast-P2A-mScarlet	Blast-P2A-mScarlet	http://vectors.intr.es/?k=pAC10108
109	pAC10109	pAAVS1-Nst-EF1aHygro2ArtTA3(-)_TetO-Blast(1-102)_NpuDnaE(N)-P2A-EGFP	Blast(1-102)-NpuDnaE(N)-P2A-EGFP	http://vectors.intr.es/?k=pAC10109
110	pAC10110	pAAVS1-Nst-EF1aHygro2ArtTA3(-)_TetO-NpuDnaE(C)_Blast(103-140)-P2A-mScarlet	NpuDnaE(C)-Blast(103-140)-P2A-mScarlet	http://vectors.intr.es/?k=pAC10110
111	pAC10111	pAAVS1-Nst-EF1aBlast2ArtTA3(-)_TetO-Hygro-P2A-NTR-E2A-	Hygro-P2A-NTR-E2A-EGFP	http://vectors.intr.es/?k=pAC10111

		EGFP		
112	pAC10112	pAAVS1-Nst-EF1aBlast2ArtTA3(-)_TetO-Hygro-P2A-NTR-E2A-mCherry	Hygro-P2A-NTR-E2A-mCherry	http://vectors.intr.es/?k=pAC10112
113	pAC10113	pAAVS1-Nst-EF1aBlast2ArtTA3(-)_TetO- Hygro(1-89)-NpuDnaE(N)-P2A-NTR-E2A-EGFP	Hygro(1-89)-NpuDnaE(N)-P2A-NTR-E2A-EGFP	http://vectors.intr.es/?k=pAC10113
114	pAC10114	pAAVS1-Nst-EF1aBlast2ArtTA3(-)_TetO- NpuDnaE(C)-Hygro(90-341)-P2A-NTR-E2A-mCherry	NpuDnaE(C)-Hygro(90-341)-P2A-NTR-E2A-mCherry	http://vectors.intr.es/?k=pAC10114
**119**	pAC10119	pLX-Hygro(1-51)-gp411_N-IRES-TagBFP2	Hygro(1-51)-gp411_N	http://vectors.intr.es/?k=pAC10119
**120**	pAC10120	pLX-gp411_C-Hygro(52-341)-IRES-mCherry	gp411_C-Hygro(52-341)	http://vectors.intr.es/?k=pAC10120
**121**	pAC10121	pLX-Hygro(1-68)-gp411_N-IRES-TagBFP2	Hygro(1-68)-gp411_N	http://vectors.intr.es/?k=pAC10121
**122**	pAC10122	pLX-gp411_C-Hygro(69-341)-IRES-mCherry	gp411_C-Hygro(69-341)	http://vectors.intr.es/?k=pAC10122
**123**	pAC10123	pLX-Hygro(1-130)-gp411_N-IRES-TagBFP2	Hygro(1-130)-gp411_N	http://vectors.intr.es/?k=pAC10123
**124**	pAC10124	pLX-gp411_C-Hygro(131-341)-IRES-mCherry	gp411_C-Hygro(131-341)	http://vectors.intr.es/?k=pAC10124
**125**	pAC10125	pLX-Hygro(1-170)-gp411_N-IRES-TagBFP2	Hygro(1-170)-gp411_N	http://vectors.intr.es/?k=pAC10125
**126**	pAC10126	pLX-gp411_C-Hygro(171-341)-IRES-mCherry	gp411_C-Hygro(171-341)	http://vectors.intr.es/?k=pAC10126
**127**	pAC10127	pLX-Hygro(1-200)-IMPDH1_N-IRES-TagBFP2	Hygro(1-200)-IMPDH1_N	http://vectors.intr.es/?k=pAC10127
**128**	pAC10128	pLX-IMPDH1_C-Hygro(201-341)-IRES-mCherry	IMPDH1_C-Hygro(201-341)	http://vectors.intr.es/?k=pAC10128
**129**	pAC10129	pLX-Hygro(1-258)-IMPDH1_N-IRES-TagBFP2	Hygro(1-258)-IMPDH1_N	http://vectors.intr.es/?k=pAC10129
**130**	pAC10130	pLX-IMPDH1_C-Hygro(259-341)-IRES-mCherry	IMPDH1_C-Hygro(259-341)	http://vectors.intr.es/?k=pAC10130
**131**	pAC10131	pLX-Hygro(1-51)-NrdJ1_N-IRES-TagBFP2	Hygro(1-51)-NrdJ1_N	http://vectors.intr.es/?k=pAC10131
**132**	pAC10132	pLX-NrdJ1_C-Hygro(52-341)-IRES-mCherry	NrdJ1_C-Hygro(52-341)	http://vectors.intr.es/?k=pAC10132
**133**	pAC10133	pLX-Hygro(1-68)-NrdJ1_N-IRES-TagBFP2	Hygro(1-68)-NrdJ1_N	http://vectors.intr.es/?k=pAC10133
**134**	pAC10134	pLX-NrdJ1_C-Hygro(69-341)-IRES-mCherry	NrdJ1_C-Hygro(69-341)	http://vectors.intr.es/?k=pAC10134
**135**	pAC10135	pLX-Hygro(1-130)-NrdJ1_N-IRES-TagBFP2	Hygro(1-130)-NrdJ1_N	http://vectors.intr.es/?k=pAC10135
**136**	pAC10136	pLX-NrdJ1_C-Hygro(131-341)-IRES-mCherry	NrdJ1_C-Hygro(131-341)	http://vectors.intr.es/?k=pAC10136
**137**	pAC10137	pLX-Hygro(1-170)-NrdJ1_N-IRES-TagBFP2	Hygro(1-170)-NrdJ1_N	http://vectors.intr.es/?k=pAC10137
**138**	pAC10138	pLX-NrdJ1_C-Hygro(171-341)-IRES-mCherry	NrdJ1_C-Hygro(171-341)	http://vectors.intr.es/?k=pAC10138
**139**	pAC10139	pLX-Hygro(1-200)-NrdJ1_N-IRES-TagBFP2	Hygro(1-200)-NrdJ1_N	http://vectors.intr.es/?k=pAC10139

**140**	pAC10140	pLX-NrdJ1_C-Hygro(201-341)-IRES-mCherry	NrdJ1_C-Hygro(201-341)	http://vectors.intr.es/?k=pAC10140
**141**	pAC10141	pLX-Puro(1-80)-gp411_N-IRES-TagBFP2	Puro(1-80)-gp411_N	http://vectors.intr.es/?k=pAC10141
**142**	pAC10142	pLX-gp411_C-Puro(81-199)-IRES-mCherry	gp411_C-Puro(81-199)	http://vectors.intr.es/?k=pAC10142
**143**	pAC10143	pLX-Puro(1-98)-gp411_N-IRES-TagBFP2	Puro(1-98)-gp411_N	http://vectors.intr.es/?k=pAC10143
**144**	pAC10144	pLX-gp411_C-Puro(99-199)-IRES-mCherry	gp411_C-Puro(99-199)	http://vectors.intr.es/?k=pAC10144
**145**	pAC10145	pLX-Puro(1-130)-gp411_N-IRES-TagBFP2	Puro(1-130)-gp411_N	http://vectors.intr.es/?k=pAC10145
**146**	pAC10146	pLX-gp411_C-Puro(131-199)-IRES-mCherry	gp411_C-Puro(131-199)	http://vectors.intr.es/?k=pAC10146
**147**	pAC10147	pLX-Puro(1-140)-gp411_N-IRES-TagBFP2	Puro(1-140)-gp411_N	http://vectors.intr.es/?k=pAC10147
**148**	pAC10148	pLX-gp411_C-Puro(141-199)-IRES-mCherry	gp411_C-Puro(141-199)	http://vectors.intr.es/?k=pAC10148
**149**	pAC10149	pLX-Puro(1-162)-gp411_N-IRES-TagBFP2	Puro(1-162)-gp411_N	http://vectors.intr.es/?k=pAC10149
**150**	pAC10150	pLX-gp411_C-Puro(163-199)-IRES-mCherry	gp411_C-Puro(163-199)	http://vectors.intr.es/?k=pAC10150
**151**	pAC10151	pLX-Puro(1-100)-IMPDH1_N-IRES-TagBFP2	Puro(1-100)-IMPDH1_N	http://vectors.intr.es/?k=pAC10151
**152**	pAC10152	pLX-IMPDH1_C-Puro(101-199)-IRES-mCherry	IMPDH1_C-Puro(101-199)	http://vectors.intr.es/?k=pAC10152
**153**	pAC10153	pLX-Puro(1-140)-IMPDH1_N-IRES-TagBFP2	Puro(1-140)-IMPDH1_N	http://vectors.intr.es/?k=pAC10153
**154**	pAC10154	pLX-IMPDH1_C-Puro(141-199)-IRES-mCherry	IMPDH1_C-Puro(141-199)	http://vectors.intr.es/?k=pAC10154
**155**	pAC10155	pLX-Puro(1-80)-NrdJ1_N-IRES-TagBFP2	Puro(1-80)-NrdJ1_N	http://vectors.intr.es/?k=pAC10155
**156**	pAC10156	pLX-NrdJ1_C-Puro(81-199)-IRES-mCherry	NrdJ1_C-Puro(81-199)	http://vectors.intr.es/?k=pAC10156
**157**	pAC10157	pLX-Puro(1-98)-NrdJ1_N-IRES-TagBFP2	Puro(1-98)-NrdJ1_N	http://vectors.intr.es/?k=pAC10157
**158**	pAC10158	pLX-NrdJ1_C-Puro(99-199)-IRES-mCherry	NrdJ1_C-Puro(99-199)	http://vectors.intr.es/?k=pAC10158
**159**	pAC10159	pLX-Puro(1-100)-NrdJ1_N-IRES-TagBFP2	Puro(1-100)-NrdJ1_N	http://vectors.intr.es/?k=pAC10159
**160**	pAC10160	pLX-NrdJ1_C-Puro(101-199)-IRES-mCherry	NrdJ1_C-Puro(101-199)	http://vectors.intr.es/?k=pAC10160
**161**	pAC10161	pLX-Puro(1-80)-gp418_N-IRES-TagBFP2	Puro(1-80)-gp418_N	http://vectors.intr.es/?k=pAC10161
**162**	pAC10162	pLX-gp418_C-Puro(81-199)-IRES-mCherry	gp418_C-Puro(81-199)	http://vectors.intr.es/?k=pAC10162
**163**	pAC10163	pLX-Puro(1-98)-gp418_N-IRES-TagBFP2	Puro(1-98)-gp418_N	http://vectors.intr.es/?k=pAC10163
**164**	pAC10164	pLX-gp418_C-Puro(99-199)-IRES-mCherry	gp418_C-Puro(99-199)	http://vectors.intr.es/?k=pAC10164
**165**	pAC10165	pLX-Puro(1-100)-gp418_N-IRES-TagBFP2	Puro(1-100)-gp418_N	http://vectors.intr.es/?k=pAC10165

**166**	pAC10166	pLX-gp418_C-Puro(101-199)-IRES-mCherry	gp418_C-Puro(101-199)	http://vectors.intr.es/?k=pAC10166
**167**	pAC10167	pLX-Puro(1-140)-gp418_N-IRES-TagBFP2	Puro(1-140)-gp418_N	http://vectors.intr.es/?k=pAC10167
**168**	pAC10168	pLX-gp418_C-Puro(141-199)-IRES-mCherry	gp418_C-Puro(141-199)	http://vectors.intr.es/?k=pAC10168
**169**	pAC10169	pLX-Puro(1-162)-gp418_N-IRES-TagBFP2	Puro(1-162)-gp418_N	http://vectors.intr.es/?k=pAC10169
**170**	pAC10170	pLX-gp418_C-Puro(163-199)-IRES-mCherry	gp418_C-Puro(163-199)	http://vectors.intr.es/?k=pAC10170
**171**	pAC10171	pLX-DEST-IRES-Hygro(1-51)-NrdJ1_N	Hygro(1-51)-NrdJ1_N	http://vectors.intr.es/?k=pAC10171
**172**	pAC10172	pLX-DEST-IRES-NrdJ1_C-Hygro(52-341)	NrdJ1_C-Hygro(52-341)	http://vectors.intr.es/?k=pAC10172
**173**	pAC10173	pLX-TagBFP2-IRES-Hygro(1-51)-NrdJ1_N	Hygro(1-51)-NrdJ1_N	http://vectors.intr.es/?k=pAC10173
**174**	pAC10174	pLX-mCherry-IRES-NrdJ1_C-Hygro(52-341)	NrdJ1_C-Hygro(52-341)	http://vectors.intr.es/?k=pAC10174
**175**	pAC10175	pLX-DEST-IRES-Hygro(1-68)-gp411_N	Hygro(1-68)-gp411_N	http://vectors.intr.es/?k=pAC10175
**176**	pAC10176	pLX-DEST-IRES-gp411_C-Hygro(69-341)	gp411_C-Hygro(69-341)	http://vectors.intr.es/?k=pAC10176
**177**	pAC10177	pLX-TagBFP2-IRES-Hygro(1-68)-gp411_N	Hygro(1-68)-gp411_N	http://vectors.intr.es/?k=pAC10177
**178**	pAC10178	pLX-mCherry-IRES-gp411_C-Hygro(69-341)	gp411_C-Hygro(69-341)	http://vectors.intr.es/?k=pAC10178
**179**	pAC10179	pLX-DEST-IRES-Hygro(1-68)-NrdJ1_N	Hygro(1-68)-NrdJ1_N	http://vectors.intr.es/?k=pAC10179
**180**	pAC10180	pLX-DEST-IRES-NrdJ1_C-Hygro(69-341)	NrdJ1_C-Hygro(69-341)	http://vectors.intr.es/?k=pAC10180
**181**	pAC10181	pLX-TagBFP2-IRES-Hygro(1-68)-NrdJ1_N	Hygro(1-68)-NrdJ1_N	http://vectors.intr.es/?k=pAC10181
**182**	pAC10182	pLX-mCherry-IRES-NrdJ1_C-Hygro(69-341)	NrdJ1_C-Hygro(69-341)	http://vectors.intr.es/?k=pAC10182
**183**	pAC10183	pLX-DEST-IRES-Hygro(1-170)-gp411_N	Hygro(1-170)-gp411_N	http://vectors.intr.es/?k=pAC10183
**184**	pAC10184	pLX-DEST-IRES-gp411_C-Hygro(171-341)	gp411_C-Hygro(171-341)	http://vectors.intr.es/?k=pAC10184
**185**	pAC10185	pLX-TagBFP2-IRES-Hygro(1-170)-gp411_N	Hygro(1-170)-gp411_N	http://vectors.intr.es/?k=pAC10185
**186**	pAC10186	pLX-mCherry-IRES-gp411_C-Hygro(171-341)	gp411_C-Hygro(171-341)	http://vectors.intr.es/?k=pAC10186
**187**	pAC10187	pLX-DEST-IRES-Hygro(1-200)-NrdJ1_N	Hygro(1-200)-NrdJ1_N	http://vectors.intr.es/?k=pAC10187
**188**	pAC10188	pLX-DEST-IRES-NrdJ1_C-Hygro(201-341)	NrdJ1_C-Hygro(201-341)	http://vectors.intr.es/?k=pAC10188
**189**	pAC10189	pLX-TagBFP2-IRES-Hygro(1-200)-NrdJ1_N	Hygro(1-200)-NrdJ1_N	http://vectors.intr.es/?k=pAC10189
**190**	pAC10190	pLX-mCherry-IRES-NrdJ1_C-Hygro(201-341)	NrdJ1_C-Hygro(201-341)	http://vectors.intr.es/?k=pAC10190
**191**	pAC10191	pLX-DEST-IRES-Puro(1-98)-gp411_N	Puro(1-98)-gp411_N	http://vectors.intr.es/?k=pAC10191

**192**	pAC10192	pLX-DEST-IRES-gp411_C-Hygro(99-199)	gp411_C-Hygro(99-199)	http://vectors.intr.es/?k=pAC10192
**193**	pAC10193	pLX-TagBFP2-IRES-Puro(1-98)-gp411_N	Puro(1-98)-gp411_N	http://vectors.intr.es/?k=pAC10193
**194**	pAC10194	pLX-mCherry-IRES-gp411_C-Hygro(99-199)	gp411_C-Hygro(99-199)	http://vectors.intr.es/?k=pAC10194
**195**	pAC10195	pLX-DEST-IRES-Puro(1-98)-NrdJ1_N	Puro(1-98)-NrdJ1_N	http://vectors.intr.es/?k=pAC10195
**196**	pAC10196	pLX-DEST-IRES-NrdJ1_C-Hygro(99-199)	NrdJ1_C-Hygro(99-199)	http://vectors.intr.es/?k=pAC10196
**197**	pAC10197	pLX-TagBFP2-IRES-Puro(1-98)-NrdJ1_N	Puro(1-98)-NrdJ1_N	http://vectors.intr.es/?k=pAC10197
**198**	pAC10198	pLX-mCherry-IRES-NrdJ1_C-Hygro(99-199)	NrdJ1_C-Hygro(99-199)	http://vectors.intr.es/?k=pAC10198
**199**	pAC10199	pLX-DEST-IRES-Hygro	Hygro	http://vectors.intr.es/?k=pAC10199
**200**	pAC10200	pLX-TagBFP2-IRES-Hygro	Hygro	http://vectors.intr.es/?k=pAC10200
**201**	pAC10201	pLX-mCherry-IRES-Hygro	Hygro	http://vectors.intr.es/?k=pAC10201
**202**	pAC10202	pLX-DEST-IRES-Puro	Puro	http://vectors.intr.es/?k=pAC10202
**203**	pAC10203	pLX-TagBFP2-IRES-Puro	Puro	http://vectors.intr.es/?k=pAC10203
**204**	pAC10204	pLX-mCherry-IRES-Puro	Puro	http://vectors.intr.es/?k=pAC10204
**205**	pAC10205	pLX-NLSGFP-IRES-Puro(1-98)-NrdJ1_N	Puro(1-98)-NrdJ1_N	http://vectors.intr.es/?k=pAC10205
**206**	pAC10206	pLX-iFP4.1-IRES-NrdJ1_C-Hygro(99-199)	NrdJ1_C-Hygro(99-199)	http://vectors.intr.es/?k=pAC10206
**207**	pAC10207	pLX-NLSGFP-IRES-Puro(1-119)-NpuDnaE(N)	Puro(1-119)-NpuDnaE(N)	http://vectors.intr.es/?k=pAC10207
**208**	pAC10208	pLX-iFP4.1-IRES-NpuDnaE(C)-Puro(120-199)	NpuDnaE(C)-Puro(120-199)	http://vectors.intr.es/?k=pAC10208
**209**	pAC10209	pLX-NLSGFP-IRES-Hygro(1-68)-NrdJ1_N	Hygro(1-68)-NrdJ1_N	http://vectors.intr.es/?k=pAC10209
**210**	pAC10210	pLX-iFP4.1-IRES-NrdJ1_C-Hygro(69-341)	NrdJ1_C-Hygro(69-341)	http://vectors.intr.es/?k=pAC10210
**211**	pAC10211	pLX-NLSGFP-IRES-Hygro(1-68)-gp411_N	Hygro(1-68)-gp411_N	http://vectors.intr.es/?k=pAC10211
**212**	pAC10212	pLX-iFP4.1-IRES-gp411_C-Hygro(69-341)	gp411_C-Hygro(69-341)	http://vectors.intr.es/?k=pAC10212
**213**	pAC10213	pLX-gp411_C-Hygro(52-89)-NpuDnaE_N-IRES-GFP	gp411_C-Hygro(52-89)-NpuDnaE_N	http://vectors.intr.es/?k=pAC10213
**214**	pAC10214	pLX-NpuDnaE_C-Hygro(90-130)-NrdJ1_N-IRES-GFP	NpuDnaE_C-Hygro(90-130)-NrdJ1_N	http://vectors.intr.es/?k=pAC10214
**215**	pAC10215	pLX-NrdJ1_C-Hygro(131-200)-SspDnaB_N-IRES-GFP	NrdJ1_C-Hygro(131-200)-SspDnaB_N	http://vectors.intr.es/?k=pAC10215
**216**	pAC10216	pLX-SspDnaB_C-Hygro(201-258)-IMPDH1_N-IRES-GFP	SspDnaB_C-Hygro(201-258)-IMPDH1_N	http://vectors.intr.es/?k=pAC10216
**217**	pAC10217	pmax-3xFLAG-Hygro-HA	3xFLAG-Hygro-HA	http://vectors.intr.es/?k=pAC10217
**218**	pAC10218	pmax-Hygro(1-171)-NpuDnaE(N)	Hygro(1-171)-NpuDnaE(N)	http://vectors.intr.es/?k=pAC10218
**219**	pAC10219	pmax-NpuDnaE(C)-Hygro(^C;172-341)	NpuDnaE(C)-Hygro(^C;172-341)	http://vectors.intr.es/?k=pAC10219
**220**	pAC10220	pmax-NpuDnaE(C)-	NpuDnaE(C)-	http://vectors.intr.es/?k=pAC10220

		Hygro(noC;172-341)	Hygro(noC;172-341)	
**221**	pAC10221	pmax-3xFLAG-Hygro(1-170)-gp411_N	3xFLAG-Hygro(1-170)-gp411_N	http://vectors.intr.es/?k=pAC10221
**222**	pAC10222	pmax-gp411_C-Hygro(171-341)-HA	gp411_C-Hygro(171-341)-HA	http://vectors.intr.es/?k=pAC10222
**223**	pAC10223	pmax-3xFLAG-Hygro(1-170)-NrdJ1_N	3xFLAG-Hygro(1-170)-NrdJ1_N	http://vectors.intr.es/?k=pAC10223
**224**	pAC10224	pmax-NrdJ1_C-Hygro(171-341)-HA	NrdJ1_C-Hygro(171-341)-HA	http://vectors.intr.es/?k=pAC10224
**225**	pAC10225	pmax-3xFLAG-Hygro(1-52)-NpuDnaE(N)	3xFLAG-Hygro(1-52)-NpuDnaE(N)	http://vectors.intr.es/?k=pAC10225
**226**	pAC10226	pmax-NpuDnaE(C)-Hygro(53-341)-HA	NpuDnaE(C)-Hygro(53-341)-HA	http://vectors.intr.es/?k=pAC10226
**227**	pAC10227	pmax-3xFLAG-Hygro(1-68)-NpuDnaE(N)	3xFLAG-Hygro(1-68)-NpuDnaE(N)	http://vectors.intr.es/?k=pAC10227
**228**	pAC10228	pmax-NpuDnaE(C)-Hygro(^C;69-341)-HA	NpuDnaE(C)-Hygro(^C;69-341)-HA	http://vectors.intr.es/?k=pAC10228
**229**	pAC10229	pmax-NpuDnaE(C)-Hygro(noC;69-341)-HA	NpuDnaE(C)-Hygro(noC;69-341)-HA	http://vectors.intr.es/?k=pAC10229
**230**	pAC10230	pmax-3xFLAG-Hygro(1-89)-NpuDnaE(N)	3xFLAG-Hygro(1-89)-NpuDnaE(N)	http://vectors.intr.es/?k=pAC10230
**231**	pAC10231	pmax-NpuDnaE(C)-Hygro(90-341)-HA	NpuDnaE(C)-Hygro(90-341)-HA	http://vectors.intr.es/?k=pAC10231
**232**	pAC10232	pmax-3xFLAG-Hygro(1-131)-NpuDnaE(N)	3xFLAG-Hygro(1-131)-NpuDnaE(N)	http://vectors.intr.es/?k=pAC10232
**233**	pAC10233	pmax-NpuDnaE(C)-Hygro(^C;132-341)-HA	NpuDnaE(C)-Hygro(^C;132-341)-HA	http://vectors.intr.es/?k=pAC10233
**234**	pAC10234	pmax-NpuDnaE(C)-Hygro(noC;132-341)-HA	NpuDnaE(C)-Hygro(noC;132-341)-HA	http://vectors.intr.es/?k=pAC10234
