# Supplementary material for: Split selectable markers
Source: Nat Commun. 2019 Oct 31;10:4968. doi: 10.1038/s41467-019-12891-2 (PMC6823381; doi:10.1038/s41467-019-12891-2)
Supplement: Supplementary file 3 — Reporting Summary [file 41467_2019_12891_MOESM3_ESM.pdf]

nature research

Corresponding author(s): Albert Cheng

Last updated by author(s): Sep 26, 2019

Reporting Summary

Nature Research wishes to improve the reproducibility of the work that we publish. This form provides structure for consistency and transparency in reporting. For further information on Nature Research policies, see Authors & Referees and the Editorial Policy Checklist.

Statistics

For all statistical analyses, confirm that the following items are present in the figure legend, table legend, main text, or Methods section.

n/a	Confirmed	
☒	☐	The exact sample size (*n*) for each experimental group/condition, given as a discrete number and unit of measurement
☐	☒	A statement on whether measurements were taken from distinct samples or whether the same sample was measured repeatedly
☐	☒	The statistical test(s) used AND whether they are one- or two-sided<br>*Only common tests should be described solely by name; describe more complex techniques in the Methods section.*
☒	☐	A description of all covariates tested
☒	☐	A description of any assumptions or corrections, such as tests of normality and adjustment for multiple comparisons
☒	☐	A full description of the statistical parameters including central tendency (e.g. means) or other basic estimates (e.g. regression coefficient) AND variation (e.g. standard deviation) or associated estimates of uncertainty (e.g. confidence intervals)
☒	☐	For null hypothesis testing, the test statistic (e.g. *F*, *t*, *r*) with confidence intervals, effect sizes, degrees of freedom and *P* value noted<br>*Give P values as exact values whenever suitable.*
☒	☐	For Bayesian analysis, information on the choice of priors and Markov chain Monte Carlo settings
☒	☐	For hierarchical and complex designs, identification of the appropriate level for tests and full reporting of outcomes
☒	☐	Estimates of effect sizes (e.g. Cohen's *d*, Pearson's *r*), indicating how they were calculated

Our web collection on statistics for biologists contains articles on many of the points above.

Software and code

Policy information about availability of computer code

Data collection	FACSDiVa software (Version 8) for flow cytometry
Data analysis	Prism 7 was used to plot graphs

For manuscripts utilizing custom algorithms or software that are central to the research but not yet described in published literature, software must be made available to editors/reviewers. We strongly encourage code deposition in a community repository (e.g. GitHub). See the Nature Research guidelines for submitting code & software for further information.

Data

Policy information about availability of data

All manuscripts must include a data availability statement. This statement should provide the following information, where applicable:

- Accession codes, unique identifiers, or web links for publicly available datasets
- A list of figures that have associated raw data
- A description of any restrictions on data availability

The data that support the findings of this study are available from the corresponding author upon reasonable request. The source data underlying Fig 4c and Supplementary Fig S1b, Fig S14b as well as raw plot numbers are provided as a Source Data file.

Field-specific reporting

Please select the one below that is the best fit for your research. If you are not sure, read the appropriate sections before making your selection.

☒ Life sciences ☐ Behavioural & social sciences ☐ Ecological, evolutionary & environmental sciences

For a reference copy of the document with all sections, see nature.com/documents/nr-reporting-summary-flat.pdf

Life sciences study design

All studies must disclose on these points even when the disclosure is negative.

Sample size	Experiments were performed in duplicate, triplicate or quadruplicate. Please refer to figure and legends.
Data exclusions	No data were excluded
Replication	Experiments were performed in duplicate, triplicate or quadruplicate. Please refer to figure and legends.
Randomization	Samples were not allocated into groups, thus randomization is not relevant for this study.
Blinding	Blinding is not relevant for this study since there was no group allocation

Reporting for specific materials, systems and methods

We require information from authors about some types of materials, experimental systems and methods used in many studies. Here, indicate whether each material, system or method listed is relevant to your study. If you are not sure if a list item applies to your research, read the appropriate section before selecting a response.

Materials & experimental systems

n/a	Involved in the study	
☐	☒	Antibodies
☐	☒	Eukaryotic cell lines
☒	☐	Palaeontology
☒	☐	Animals and other organisms
☒	☐	Human research participants
☒	☐	Clinical data

Methods

n/a	Involved in the study	
☒	☐	ChIP-seq
☐	☒	Flow cytometry
☒	☐	MRI-based neuroimaging

Antibodies

Antibodies used	HA-epitope (#3724, Cell Signaling); FLAG-epitope (#F7425, Millipore Sigma); Vinculin (EPR8185, Abcam)
Validation	Validation of antibodies are provided on manufacturer's websites

Eukaryotic cell lines

Policy information about cell lines

Cell line source(s)	HEK293T, U2OS, HeLa (ATCC); KOLF2_C1 (Bill Skarnes, subcloned from HipSci KOLF2)
Authentication	None of the cell lines used were authenticated
Mycoplasma contamination	Cell lines were not tested for mycoplasma contaimination
Commonly misidentified lines<br>(See ICLAC register)	N/A

Flow Cytometry

Plots

Confirm that:

☒ The axis labels state the marker and fluorochrome used (e.g. CD4-FITC).

☒ The axis scales are clearly visible. Include numbers along axes only for bottom left plot of group (a 'group' is an analysis of identical markers).

☒ All plots are contour plots with outliers or pseudocolor plots.

☒ A numerical value for number of cells or percentage (with statistics) is provided.

Methodology

Sample preparation	Cells were trypsinized, suspended in media then analyzed on a LSRFortessa X-20 or FACSymphony flow cytometers (BD Bioscience)
Instrument	LSRFortessa X-20 or FACSymphony flow cytometers (BD Bioscience)
Software	FACSDiVa software (Version 8)
Cell population abundance	N/A
Gating strategy	Boundaries between positive and negative populations were defined by running a negative sample.

☒ Tick this box to confirm that a figure exemplifying the gating strategy is provided in the Supplementary Information.
